# Comparison of percutaneous vs. cutdown access for endovascular aortic repair in the treatment of type B aortic dissection: a meta-analysis

**DOI:** 10.3389/fcvm.2025.1673817

**Published:** 2025-11-24

**Authors:** Zhongyou Liang, Ziping Huang, Minyao Wu, Lidai Lin, Binyao Chen, Shan Yin, Huaicheng Li, Jieru Quan, Weiming Liang, Xiaolong Huang

**Affiliations:** 1 The First Affiliated Hospital of Guangxi University of Science and Technology, Guangxi University of Science and Technology, Liuzhou, Guangxi, China; 2 School of Economics and Management, Guangxi University of Science and Technology, Liuzhou, Guangxi, China

**Keywords:** aortic dissection, endovascular aneurysm repair, percutaneous, cutdown, pseudoaneurysm, meta-analysis

## Abstract

**Introduction:**

This meta-analysis was designed to compare the safety and efficacy of percutaneous endovascular aortic repair (PEVAR) with endovascular aortic repair by cutdown access (CEVAR) in the treatment of TBAD.

**Materials and methods:**

Four databases (PubMed, Embase, Web of Science, and Cochrane Library) were carefully queried for articles comparing PEVAR and CEVAR in patients with TBAD. The search was performed from the foundation of the databases until March 31, 2025.

**Results:**

Totally 24 studies were included in this meta-analysis. The meta-analysis included a group of 28,263 patients diagnosed with TBAD, with 14,534 patients undergoing PEVAR and 13,729 patients undergoing CEVAR. In comparison to CEVAR, PEVAR resulted in a reduced hospital length of stay (MD = −2.16 days, 95% CI: −3.05 to −1.27, *P* < 0.00001), decreased operative time (MD = −40.87 min, 95% CI: −49.72 to −32.02, *P* < 0.00001), shorter postoperative duration (MD = −1.01 days, 95% CI: −1.56 to −0.45, *P* = 0.0004), diminished incidence of groin infection (OR = 0.44, 95% CI: 0.30 to 0.65, *P* < 0.0001), lower occurrence of heart-related complications (OR = 0.76, 95% CI: 0.59 to 1.00, *P* = 0.05), and reduced incidence of lymphocele (OR = 0.49, 95% CI: 0.24 to 0.98, *P* = 0.04), but a higher incidence of surgical suture failure (OR = 2.61, 95% CI: 1.52 to 4.50, *P* = 0.0005) and pseudoaneurysm (OR = 2.64, 95% CI: 1.09 to 6.41, *P* = 0.03). No statistically significant differences were observed between the two groups concerning estimated blood loss, ICU admissions, hematoma, acute kidney injury, lower extremity revascularization, ischemic colitis, and deep venous thrombosis.

**Conclusions:**

Compared to CEVAR, PEVAR was associated with a shorter hospital stay, reduced operative time, quicker postoperative recovery, lower rates of groin infections, fewer cardiac complications, and a diminished occurrence of lymphocele; however, it exhibited a higher incidence of pseudoaneurysm and an increased rate of surgical suture failure. PEVAR was a safe and effective method for the treatment of TBAD.

**Systematic Review Registration:**

https://www.crd.york.ac.uk/prospero/display_record.php?ID=CRD420251110307, PROSPERO CRD420251110307.

## 1 Introduction

Aortic dissection is the predominant acute aortic syndrome (1). Aortic dissection affects four to five individuals per 100,000 each year, rendering it the most prevalent aortic-specific emergency, with 20%–30% of patients succumbing prior to hospital transfer (2). It is typically categorized into two categories based on anatomical characteristics: Stanford type B dissections commence distal to the ostium of the left subclavian artery, whereas Stanford type A dissections involve the ascending aorta and the aortic arch (3). Due to advancements in interventional therapy techniques, the evolution of related medical equipment, and the consolidation of surgical expertise, endovascular repair utilizing covered stents has become the predominant treatment for aortic dissection. This technique is preferred for its less invasive characteristics and positive prognosis, especially in cases of Stanford type B aortic dissection, where it has emerged as the favored surgical treatment (4).

Surgeons frequently select the femoral artery as the ideal access point for endovascular repair of dissection. Two independent surgical techniques exist: one use a typical incision to carefully dissect the layers of the femoral artery. Nonetheless, the CEVAR procedure requires a surgical incision of roughly 5 cm in the inguinal region, and is influenced by factors such as the postoperative environment, care, and alterations to the incision dressing, resulting in a relatively heightened risk of infection (5). The alternative procedure entails percutaneous puncture to access the femoral artery, which has increasingly been the dominant way in recent years, despite contemporary breakthroughs in medical and surgical interventions (6). The endovascular repair of abdominal and/or thoracic aortic disease, coupled with the progression of endograft technology that facilitates lower profiles and smaller access sheaths, has prompted the majority of vascular surgeons to adopt an endovascular-first strategy over an open-first approach. Technological advancements in the field have expanded the criteria of suitable aortic anatomy for secure endovascular repair (7). Totally PEVARP was first introduced in 1999 (8), and referred to the practice of CFA cannulation site closure using a “preclose” technique with a Perclose ProGlide® (Abbott Vascular) closure device.Since that time, this technique has been widely adopted (9). PEVAR is technically viable, alleviates patient discomfort, reduces operative duration, shortens hospital stays, diminishes complication rates, and decreases the ambulation period associated with the percutaneous method (10, 11). However, percutaneous access may result in injury to the femoral artery, potentially causing hematoma, pseudoaneurysm development, and arterial dissection (12). In addition, a financial drawback of percutaneous access has been noted due to PEVAR's dependence on expensive closure devices (6).

Therefore, Controversy persists on which of the two techniques possesses a definite benefit. A prior meta-analysis demonstrated no statistically significant differences between the two surgical techniques regarding overall hospitalization expenditures, length of hospital stay, postoperative hematoma, femoral artery stenosis or occlusion, and other problems (13). Moreover, the elevated puncture site complicates bleeding management using puncture suture methods, resulting in a greater frequency of postoperative pseudoaneurysms compared to incision surgery (14). In recent years, a growing number of clinical research articles have been published comparing the two surgical techniques (15, 16). Consequently, it is essential to perform an updated meta-analysis to assess the comparative advantages of the two surgical techniques. This meta-analysis was designed to compare the safety and efficacy of PEVAR with CEVAR in the treatment of TBAD.

## 2 Materials and methods

### 2.1 Search strategy

This meta-analysis followed the 2020 guidelines established by the Preferred Reporting Project for Systematic Review and Meta-Analysis (PRISMA).The study had been formally registered at PROSPERO with the registration number CRD420251110307. A comprehensive search was performed in four databases, including PubMed, Embase, Web of Science, and the Cochrane Library, to collect literature published up until March 31, 2025. The search methodology adhered to the PICOS principle and utilised a combination of MeSH terms and unrestricted text phrases. The search strategy employed consisted of combining the keywords “Percutaneous”, “Cutdown”, “Aortic aneurysm repair”, and “Type B aortic dissection”. Supplementary Data Sheet 1 offered a thorough summary of the search record. In addition, we conducted a thorough manual examination of the bibliographies of the identified papers, as well as pertinent reviews and meta-analyses, in order to uncover any studies that fit the criteria for inclusion.

### 2.2 Inclusion and exclusion criteria

The criteria for inclusion were as follows: (1) patients diagnosed with type B aortic dissection; (2) patients in the intervention group received percutaneous endovascular aneurysm repair; (3) patients in the controlled group received endovascular aortic repair by cutdown access; (4) at least one of the following outcomes was reported: hospital length of stay, operative time, estimated blood loss, stay of postoperative, patients requiring ICU stay, surgical suture failure, pseudoaneurysm, hematoma, groin infection, heart-related complications, lymphocele, acute kidney injury, lower extremity revascularization, ischemic colitis and deep venous thrombosis; (5) Study types: randomised controlled trials (RCTs), retrospective studies or prospective study.

The exclusion criteria were as follows: (1) other types of articles, such as case reports, protocols, letters, editorials, comments, reviews, meta-analyses; (2) not relevant; (3) failed to obtain full-text; (4) data cannot be extracted; (5) duplicate patient cohort. If there were studies conducted in the same location and population at different times, only the largest sample size or most recent studies would be included.

### 2.3 Selection of studies

The literature selection procedure, which included the elimination of duplicate entries, was carried out using EndNote (Version 20; Clarivate Analytics). The initial search was conducted by two autonomous reviewers. The redundant items were removed, and the titles and abstracts were evaluated to determine their relevance. Subsequently, each study was classified as either included or omitted. The settlement was reached through consensus. In the event that the parties involved are unable to come to a mutual agreement, a third reviewer assumes the function of a mediator.

### 2.4 Data extraction

Two independent reviewers extracted data. The extracted data included: (1) basic characteristics of studies included: author, nationality, year of publication; (2) baseline characteristics of study subjects: age, sample size, access sites; (3) outcome indicators: hospital length of stay, operative time, estimated blood loss, stay of postoperative, patients requiring ICU stay, surgical suture failure, pseudoaneurysm, hematoma, groin infection, heart-related complications, lymphocele, acute kidney injury, lower extremity revascularization, ischemic colitis, deep venous thrombosis.

### 2.5 Quality assessment

Two independent reviewers assessed the quality assessment of the studies that were included. In this study, we utilised the Newcastle-Ottawa Scale (NOS) (17) to assess the quality of retrospective or prospective cohort studies, including eight domains: (1) representativeness of the exposed cohort; (2) selection of the non-exposed cohort; (3) ascertainment of exposure; (4) demonstration that outcome of interest was not present at start of study; (5) comparability of cohorts on the basis of the design or analysis; (6) assessment of outcome; (7) was follow-up long enough for outcomes to occur; (8) adequacy of follow-up of cohorts. The score on this scale is considered high quality research if it was 7 stars or above. We evaluated the RCTs in this study using the risk of bias assessment tool implemented by the Cochrane Collaboration (18). The assessment encompassed seven essential domains: (1) randomization technique; (2) allocation concealment; (3) blinding of participants and treatment administrators; (4) blinding of outcome assessments; (5) completeness of outcome data; (6) selective outcome reporting; and (7) additional bias sources. All studies were evaluated as being at risk in each of the seven domains. Each study was categorized as high-quality, medium-quality, or low-quality based on the evaluation results according to the specified criteria.

### 2.6 Statistical analysis

The results were analysed using Review Manager 5.3, a software developed by the Cochrane Collaboration in Oxford, UK. The continuous variables were compared using the weighted mean difference (WMD) and a 95% confidence interval (CI). The odds ratio (OR) was used to compare binary variables, along with a 95% CI. The medians and interquartile ranges of continuous data were converted to the mean and standard deviation. The Cochrane's Q test and the *I*^2^ index were used to evaluate the statistical heterogeneity among the studies included. The Cochrane Q *p* value and I^2^ statistic were used to evaluate the heterogeneity of each meta-analysis. A fixed-effect model (FEM) was used for low heterogeneity (I^2^ < 50%), and a random-effect model (REM) was used for high heterogeneity (I^2^ ≥ 50%) when analyzing pooled data. A *p*-value below 0.05 was considered to have statistical significance. Funnel plots were used to evaluate the publication bias.

## 3 Results

### 3.1 Search results

**Figure 1:**
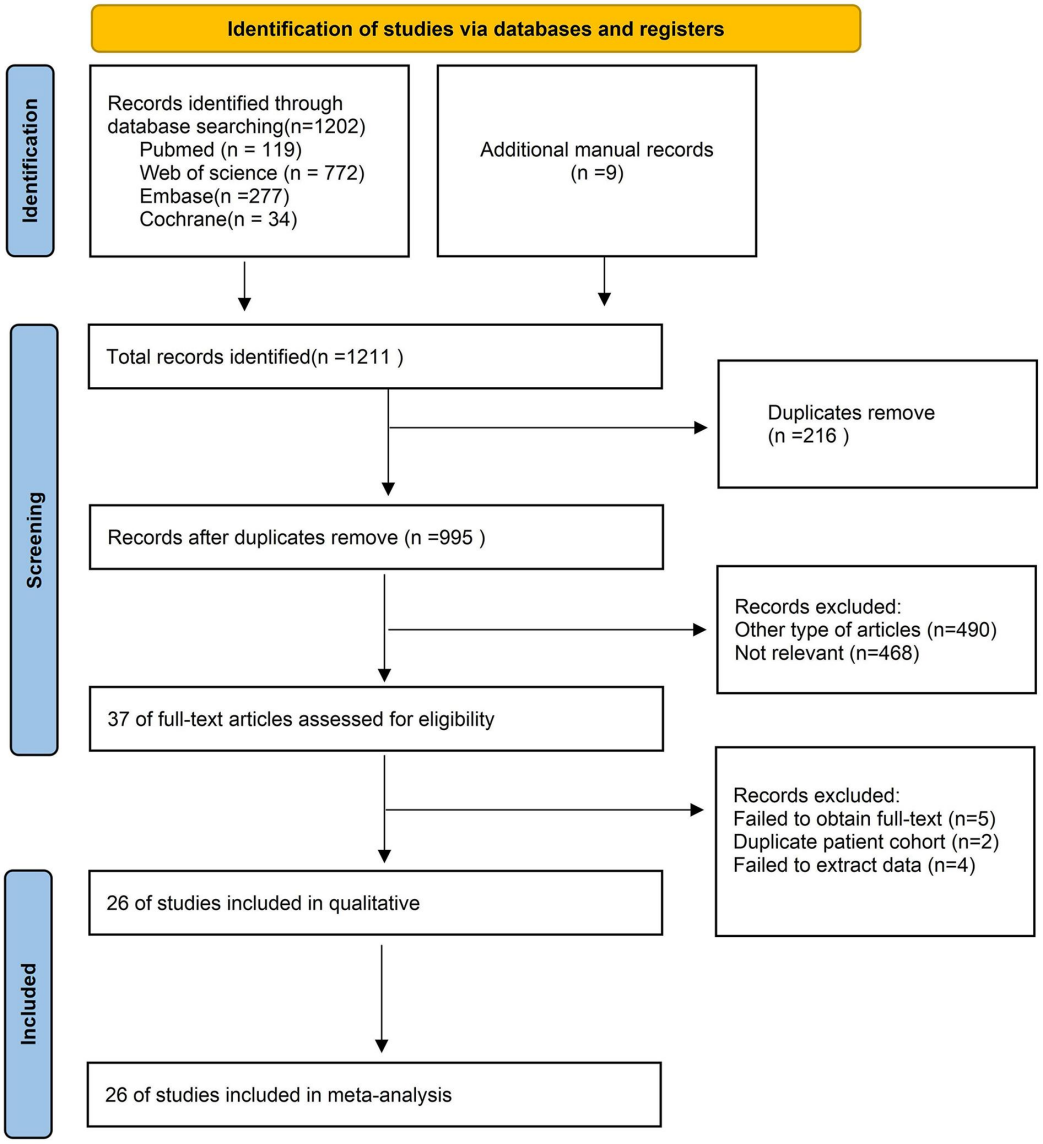
Flow chart of literature search strategies.

 Figure 1 illustrated the process of selecting and incorporating articles. A grand total of 1,211 publications were acquired from a combination of four databases and additional manual records. After applying the predetermined criteria for inclusion and exclusion, a total of 24 articles (6, 7, 10, 11, 15, 16, 19–38) were selected for the final meta-analysis.

### 3.2 Characteristics of included studies and patients

The meta-analysis included a total of 24 studies (6, 7, 10, 11, 15, 16, 19–38), which consisted of 4 RCTs, 2 prospective studies and 18 retrospective studies. The meta-analysis included a combined total of 28,263 participants, with 14,534 patients undergoing PEVAR and 13,729 patients undergoing CEVAR. The studies were undertaken in several nations, including the USA, Japan, China, Turkey, Netherlands, Austria, Saudi Arabia, Finland. Table 1 contained detailed characteristics of included studies and patients.

**Table 1:** Characteristics of included studies and patients.

Author	Year	Recruitment period	Country	Type of study		Age (Mean ± SD)	Male/female	Patients/access sites	BMI
Taku (26)	2015	2009–2013	Japan	Retrospective study	PEVAR	75.5 ± 7.6	46/6	50/100	23.2 ± 2.8
					CEVAR	75.3 ± 7.9	80/16	96/192	22.7 ± 3.7
Peter (10)	2020	2005–2013	USA	Retrospective study	PEVAR	NR	NR	49/49	NR
					CEVAR	NR	NR	24/24	NR
Lin (23)	2018	2011–2016	China	Retrospective study	PEVAR	58.27 ± 11.60	51/9	60/99	NR
					CEVAR	61.11 ± 12.78	43/12	55/84	NR
Sahin (19)	2020	2014–2020	Turkey	Retrospective study	PEVAR	58.7 ± 9.9	45/11	56/56	26.4 ± 5.9
					CEVAR	57.2 ± 11.8	29/11	40/40	26.8 ± 6.1
Christopher (20)	2017	2008–2014	USA	Retrospective study	PEVAR	75.2 ± 0.85	88/14	102/204	28.4 ± 1.26
					CEVAR	74.6 ± 0.89	83/15	98/182	27.8 ± 0.57
Dipankar (21)	2017	2012–2015	USA	Retrospective study	PEVAR	73.3 ± 9.5	115/17	132/264	27.1 ± 5.0
					CEVAR	72.1 ± 8.2	45/6	51/102	27.9 ± 4.5
Dominique (22)	2015	2011–2013	Netherlands	prospective study	PEVAR	74	81/1,027	1,108/2,177	NR
					CEVAR	74	81/2,923	3,004/5,632	NR
Miriam (24)	2018	2016–2017	Austria	RCT	PEVAR	74.04	43/7	50/50	NR
					CEVAR	74.04	43/7	50/50	NR
Akbulut (27)	2022	2013–2020	Turkey	Retrospective study	PEVAR	70.5 ± 8	57/8	65/65	NR
					CEVAR	68.9 ± 7.4	78/9	87/87	NR
Weesam (28)	2012	2000–2009	USA	Retrospective study	PEVAR	78	0/31	31/24	NR
					CEVAR	75.5	0/112	112/154	NR
Altoijry (16)	2023	2015–2022	Saudi Arabia	Retrospective study	PEVAR	62.0 ± 18.5	31/4	35/35	27.7 ± 6.6
					CEVAR	57.3 ± 20.6	23/1	24/24	29.4 ± 10.5
Baxter (7)	2021	2010–2021	USA	Retrospective study	PEVAR	72.1 ± 10.2	1,687/330	2,017/2,017	27.8 ± 5.5
					CEVAR	71.4 ± 10.5	2,002/444	2,446/2,446	27.3 ± 5.1
Etezadi (30)	2011	2003–2010	USA	Retrospective study	PEVAR	75 ± 8.2	58/12	70/85	26.9 ± 3.9
					CEVAR	76.3 ± 7.7	326/49	375/557	27.2 ± 4.6
Hahl (15)	2023	2005–2013	Finland	Retrospective study	PEVAR	76.3	223/34	257/257	NR
					CEVAR	77.9	166/20	186/ 186	NR
Kauvar (32)	2016	2011–2013	USA	Retrospective study	PEVAR	74 ± 9	1,264/269	1,533/1,533	28.3 ± 5.9
					CEVAR	73 ± 9	1,285/304	1,589/1,589	28.5 ± 6.0
Siracuse (34)	2018	2014–2017	USA	Retrospective study	PEVAR	73.0 ± 8.8	6,926/1,414	8,340/8,340	28.2 ± 5.9
					CEVAR	73.5 ± 8.8	2,701/694	4,747/4,747	27.9 ± 6.0
Tay (36)	2022	2015–2021	USA	Retrospective study	PEVAR	70 ± 8	18/3	21/42	30 ± 7
					CEVAR	75 ± 10	12/2	14/28	30 ± 6
WU (38)	2022	2017–2021	China	Retrospective study	PEVAR	59	141/37	178/178	NR
					CEVAR	57	97/20	117/117	NR
Howell (31)	2002	2001–2004	USA	prospective study	PEVAR	73 ± 7.1	ND	30/60	NR
					CEVAR	ND	ND	96/96	NR
Torsello (6)	2003	2002–2002	USA	RCT	PEVAR	74.5 ± 10.4	14/1	15/25	NR
					CEVAR	71 ± 9.6	15/0	15/30	NR
Vierhout (11)	2019	2014–2016	Netherlands	RCT	PEVAR	72.6 ± 8.1	67/6	73/137	27.5 ± 3.6
					CEVAR	72.4 ± 6.2	56/8	64/137	27.2 ± 3.7
Thurston (37)	2019	2009–2016	USA	Retrospective study	PEVAR	74.0 ± 10.1	39/11	50/100	NR
					CEVAR	72.4 ± 7.5	38/12	50/100	NR
Smith (35)	2009	2005–2007	USA	Retrospective study	PEVAR	72 ± 10	19/2	22/38	27.4 ± 5
					CEVAR	71 ± 8	21/1	22/50	27.4 ± 6
Nelson (33)	2014	2010–2012	USA	RCT	PEVAR	74 ± 11	91/10	101/101	28.4 ± 4.3
					CEVAR	73 ± 8.8	45/5	50/50	28 ± 4.7

PEVAR, percutaneous endovascular aortic repair; CEVAR, endovascular aortic repair by cutdown access; NR, not reported.

### 3.3 Quality assessment

**Figure 2:**
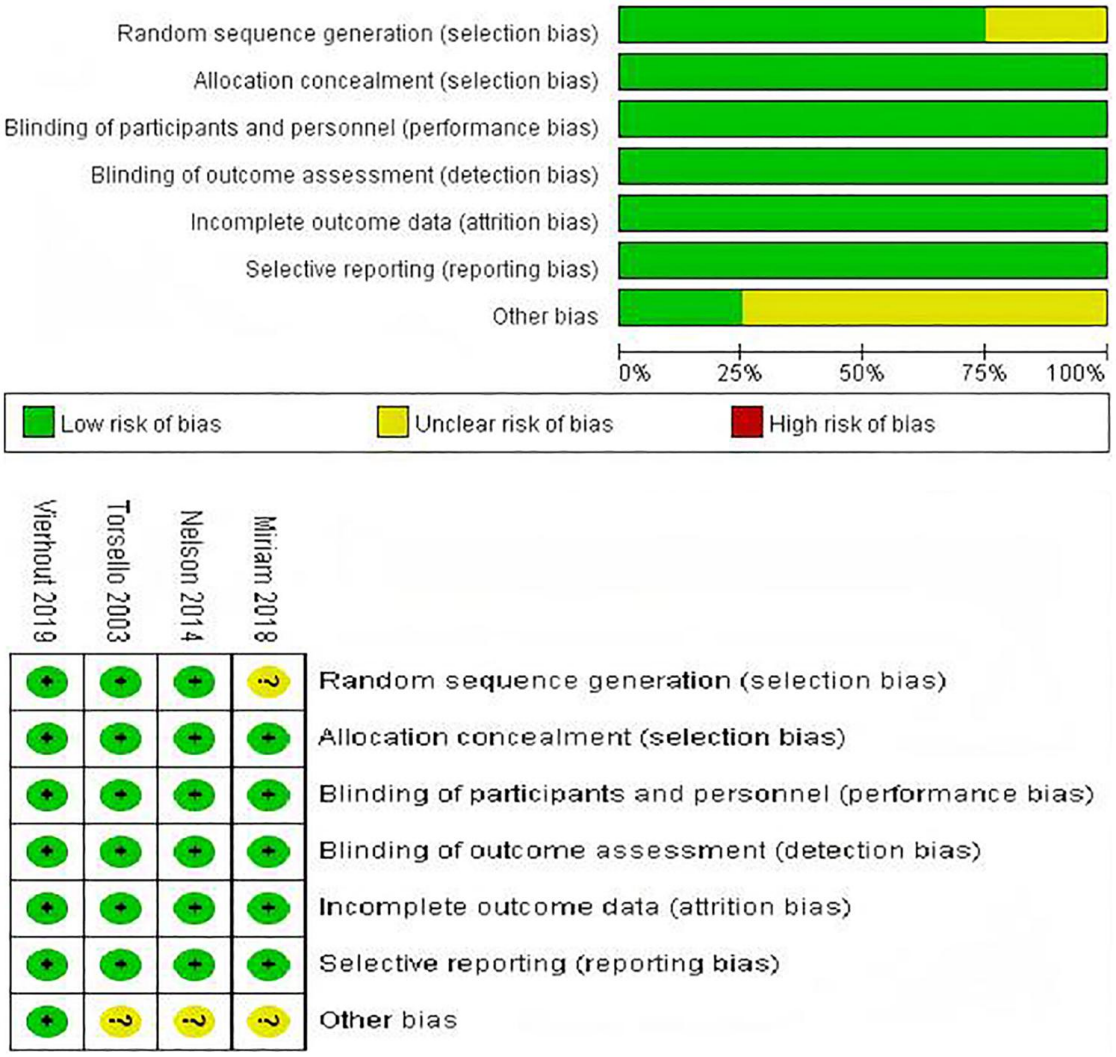
Risk of bias assessment for the included RCTs.

The Newcastle-Ottawa Scale was employed to assess the quality of the listed studies. Among the 20 studies, three studies had a rating of 9 points, twelve studies received a rating of 8 points, and nine studies received a rating of 7 points. Table 2 presented a detailed analysis of the quality assessment conducted by NOS and Jadad (39). Cochrane Risk of Bias Tool evaluation indicated that the included trials were of high quality. Three trials produced a sufficient random sequence, four studies reported appropriate allocation concealment, four studies clearly implemented participant blinding, four studies reported outcome assessor blinding, four studies provided complete outcome data, four studies did not engage in selective reporting, and one studies did not exhibit any other bias (Figure 2).

**Table 2:** Quality assessment according to the Newcastle-Ottawa scale.

Author, year	Selection				Comparability		Outcome			Total scores
	Representativeness	Selection of non-exposure	Ascertainment of exposure	Outcome not present at start	Comparability on most important factors	Comparability on other risk factors	Assessment of outcome	Adequate follow-up time	Complete follow-up	
Taku, 2015 (26)	*	–	*	*	*	*	*	*	–	7
Peter, 2020 (10)	*	*	–	*	*	*	*	*	–	7
Lin, 2018 (23)	*	*	*	*	*	*	*	*	*	9
Sahin, 2020 (19)	*	–	*	*	*	–	*	*	*	7
Christopher, 2017 (20)	*	*	–	*	*	*	*	*	–	7
Dipankar, 2017 (21)	*	*	–	*	*	*	*	*	*	8
Dominique, 2015 (22)	*	*	*	*	*	*	*	*	*	9
Akbulut, 2022 (27)	*	–	*	*	*	*	*	*	*	8
Weesam, 2012 (28)	*	*	*	*	*	*	*	–	–	7
Altoijry, 2023 (16)	*	*	*	*	*	*	*	*	–	8
Baxter, 2021 (7)	*	*	–	*	*	*	*	*	–	7
Etezadi, 2011 (30)	*	*	*	*	*	*	*	*	–	8
Hahl, 2023 (15)	*	*	*	*	*	*	*	–	–	7
Kauvar, 2016 (32)	*	*	*	*	*	*	*	*	–	8
Siracuse, 2018 (34)	*	*	*	*	*	*	*	*	*	9
Tay, 2022 (36)	*	*	*	*	*	*	*	*	–	8
WU, 2022 (38)	*	-	*	*	*	*	*	*	*	8
Howell, 2002 (31)	*	*	*	*	*	*	*	*	–	8
Thurston, 2019 (37)	*	–	*	*	*	*	*	*	–	7
Smith, 2009 (35)	*	*	*	*	*	*	*	*	–	8

"*"indicates criterion met; “–"indicates significant of criterion not.

### 3.4 Outcomes

The results of the meta-analysis were summarized in Table 3.

**Table 3:** Results of the meta-analysis.

Outcomes		Sample size	No. of studies	Heterogeneity		Overall effect size	95% CI of overall effect	*P* Value
				I^2^ (%)	*P* Value			
Hospital length of stay	PEVAR	2,729	9	92	<0.00001	WMD = −2.16	−3.05 to −1.27	<0.00001
	CEVAR	3,056						
Operative time	PEVAR	10,449	11	95	<0.00001	WMD = −40.87	−49.72 to −32.02	<0.00001
	CEVAR	6,906						
Estimated blood loss	PEVAR	8,811	6	100	<0.00001	WMD = −128.55	−277.51 to 20.40	0.09
	CEVAR	5,163						
Stay of postoperative	PEVAR	10,035	4	96	<0.00001	WMD = −1.01	−1.56 to −0.45	0.0004
	CEVAR	6,489						
Patients requiring ICU stay	PEVAR	269	3	74	0.02	OR = 0.26	0.08∼0.86	0.03
	CEVAR	173						
Surgical suture failure	PEVAR	1,731	10	48	0.04	OR = 2.61	1.52∼4.50	0.0005
	CEVAR	1,539						
Pseudoaneurysm	PEVAR	2,643	7	0	0.53	OR = −2.64	1.09∼6.41	0.03
	CEVAR	3,567						
Hematoma	PEVAR	2,804	12	0	0.46	OR = 0.81	0.49∼1.32	0.39
	CEVAR	3,734						
Groin infection	PEVAR	12,678	18	3	0.42	OR = 0.44	0.30∼0.65	<0.0001
	CEVAR	11,878						
Heart-related complications	PEVAR	12,153	7	0	0.80	OR = 0.76	0.59∼1.00	0.05
	CEVAR	10,316						
Lymphocele	PEVAR	518	7	40	0.12	OR = 0.49	0.24∼0.98	0.04
	CEVAR	444						
Acute kidney injury	PEVAR	2,944	6	0	0.79	OR = 0.88	0.53∼1.47	0.64
	CEVAR	4,732						
Lower extremity revascularization	PEVAR	12,039	11	0	0.82	OR = 1.17	0.86∼1.58	0.33
	CEVAR	10,625						
Ischemic colitis	PEVAR	1,178	3	0	0.43	OR = 1.02	0.42∼2.49	0.96
	CEVAR	3,042						
Deep venous thrombosis	PEVAR	2,807	5	63	0.03	OR = 1.14	0.34∼3.84	0.84
	CEVAR	5,219						

#### 3.4.1 Hospital length of stay

The data was provided in 9 (7, 15, 16, 19, 25–27, 29, 36–38) out of the 24 articles included. The hospital length of stay in the PEVAR group was significantly shorter than that in the CEVAR group (MD = −2.16 days, 95% CI: −3.05 to −1.27, *P* < 0.00001, I^2^ = 92%) (Supplementary Figure S1).

#### 3.4.2 Operative time

The data was provided in 11 (6, 19, 23, 25–27, 31–34, 36, 38) out of the 24 articles included. The Operative time in the PEVAR group was significantly shorter than that in the CEVAR group (MD = −40.87 min, 95% CI: −49.72 to −32.02, *P* < 0.00001, I^2^ = 95%) (Supplementary Figure S2).

#### 3.4.3 Estimated blood loss

The data was provided in 6 (20, 23, 31, 33, 34, 38) out of the 24 articles included. There was no statistical significance in the amount of estimated blood loss between the two groups (MD = −128.55 mL, 95% CI: −277.51 to 20.40, *P* = 0.09, I^2^ = 100%) (Supplementary Figure S3).

#### 3.4.4 Stay of postoperative

The data was provided in 4 (20, 23, 32, 34) out of the 24 articles included. The length of stay of postoperative in the PEVAR group was statistically shorter than that in the CEVAR group (MD = −1.01days, 95% CI: −1.56 to −0.45, *P* = 0.0004, I^2^ = 96%) (Supplementary Figure S4).

#### 3.4.5 Patients requiring ICU stay

The data was provided in 3 (16, 20, 21, 25) out of the 24 articles included. There was no significant difference in the amount of patients requiring ICU stay between the two groups (OR = 0.26, 95% CI: 0.08 to 0.86, *P* = 0.0004, I^2^ = 96%) (Supplementary Figure S5).

#### 3.4.6 Surgical suture failure

The data was provided in 10 (11, 15, 16, 19, 20, 23–26, 29, 33, 36) out of the 24 articles included. The incidence of surgical suture failure in the PEVAR group was significantly higher than that in the CEVAR group (OR = 2.61, 95% CI: 1.52 to 4.50, *P* = 0.0005, I^2^ = 48%) (Supplementary Figure S6).

#### 3.4.7 Pseudoaneurysm

The data was provided in 7 (7, 11, 15, 19, 23, 28, 30) out of the 24 articles included. The incidence of pseudoaneurysm in the PEVAR group was significantly higher than that in the CEVAR group (OR = 2.64, 95% CI: 1.09 to 6.41, *P* = 0.03, I^2^ = 0%) (Supplementary Figure S7).

#### 3.4.8 Hematoma

The data was provided in 12 (7, 11, 16, 19, 20, 23, 24, 28, 30, 35, 36, 38) out of the 24 articles included. There was no statistically significant difference in the incidence of hematoma between the two groups (OR = 0.81, 95% CI: 0.49 to 1.32, *P* = 0.39, I^2^ = 0%) (Supplementary Figure S8).

#### 3.4.9 Groin infection

The data was provided in 9 (7, 10, 11, 15, 16, 19, 20, 22–24, 26–30, 34–36, 38) out of the 24 articles included. The incidence of groin infection in the PEVAR group was significantly lower than that in the CEVAR group (OR = 0.44, 95% CI: 0.30 to 0.65, *P* < 0.0001, I^2^ = 3%) (Supplementary Figure S9).

#### 3.4.10 Heart-related complications

The data was provided in 7 (10, 20–22, 25, 29, 32, 34, 36) out of the 24 articles included. The incidence of heart-related complications in the PEVAR group was significantly lower than that in the CEVAR group (OR = 0.76, 95% CI: 0.59 to 1.00, *P* = 0.05, I^2^ = 0%) (Supplementary Figure S10).

#### 3.4.11 Lymphocele

The data was provided in 7 (6, 10, 23, 26, 27, 33, 38) out of the 24 articles included. The incidence of lymphocele in the PEVAR group was significantly lower than that in the CEVAR group (OR = 0.49, 95% CI: 0.24 to 0.98, *P* = 0.04, I^2^ = 40%) (Supplementary Figure S1^1^).

#### 3.4.12 Acute kidney injury

The data was provided in 6 (10, 21, 22, 25, 32, 33, 36) out of the 24 articles included. There was no statistically significant difference in the incidence of acute kidney injury between the two groups (OR = 0.88, 95% CI: 0.53 to 1.47, *P* = 0.64, I^2^ = 0).(Supplementary Figure S1^2^).

#### 3.4.13 Lower extremity revascularization

The data was provided in 11 (7, 10, 20–25, 27, 29, 33, 34, 36) out of the 24 articles included. There was no statistically significant difference in the incidence of lower extremity revascularization between the two groups (OR = 1.17, 95% CI: 0.86 to 1.58, *P* = 0.33, I^2^ = 0) (Supplementary Figure S13).

#### 3.4.14 Ischemic colitis

The data was provided in 3 (10, 22, 25, 29, 36) out of the 24 articles included. There was no significant difference in the incidence of ischemic colitis between the two groups (OR = 1.02, 95% CI: 0.42 to 2.49, *P* = 0.96, I^2^ = 0%) (Supplementary Figure S14).

#### 3.4.15 Deep venous thrombosis

The data was provided in 5 (22, 23, 25, 30, 32, 36) out of the 24 articles included. There was no significant difference in the incidence of deep venous thrombosis between the two groups (OR = 1.14, 95% CI: 0.34 to 3.84, *P* = 0.84, I^2^ = 63%) (Supplementary Figure S15).

### 3.5 Publication bias

Funnel plots were used to evaluate publication bias. The bilaterally symmetrical funnel plot did not provide any apparent indications of publication bias with regards to Hospital length of stay (Supplementary Figure S16), operative time (Supplementary Figure S17), groin infection (Supplementary Figure S18), lower extremity revascularization (Supplementary Figure S19). The funnel plots for surgical suture failure (Supplementary Figure S20) and hematoma (Supplementary Figure S21) indicated that the studies included in the analysis had significant asymmetry.

### 3.6 Subgroup analysis regarding studies with long term follow up

A subgroup analysis regarding studies with long term follow up (at least 6 months) was performed (Table 4; Supplementary Figure S22–S31). Compared with CEVAR, PEVAR resulted in a reduced hospital length of stay, decreased operative time, and reduced incidence of groin infection and lymphocele. No statistically significant differences were observed between the two groups concerning hematoma, acute kidney injury, lower extremity revascularization, deep venous thrombosis, surgical suture failure and pseudoaneurysm.

**Table 4:** Meta-analysis results of studies with a follow-up period of six months or more.

Outcomes		Sample size	No. of studies	Heterogeneity		Overall effect size	95% CI of overall effect	*P* Value
				I^2^ (%)	*P* Value			
Hospital length of stay	PEVAR	2,360	4	96	<0.00001	WMD = −1.42	−1.65 to −1.18	<0.00001
	CEVAR	2,732						
Operative time	PEVAR	187	3	95	<0.00001	WMD = −40.42	−50.24 to −30.60	<0.00001
	CEVAR	151						
Hematoma	PEVAR	2,161	4	0	0.82	OR = 0.41	0.15 to 1.11	0.08
	CEVAR	3,066						
Surgical suture failure	PEVAR	379	3	79	0.008	OR = 1.90	0.18∼20.03	0.59
	CEVAR	250						
Pseudoaneurysm	PEVAR	2,359	3	55	0.11	OR = 2.77	0.90∼8.48	0.07
	CEVAR	3,188						
Groin infection	PEVAR	2,483	6	37	0.16	OR = 0.41	0.18∼0.90	0.03
	CEVAR	3,339						
Lymphocele	PEVAR	166	2	0	0.80	OR = 0.11	0.01∼1.00	0.05
	CEVAR	137						
Acute kidney injury	PEVAR	122	2	0	0.83	OR = 1.79	0.35∼9.33	0.49
	CEVAR	64						
Lower extremityrevascularization	PEVAR	2,204	4	0	0.42	OR = 0.55	0.21∼1.46	0.23
	CEVAR	2,596						
Deep venous thrombosis	PEVAR	106	2	74	0.05	OR = 0.65	0.11∼3.89	0.64
	CEVAR	571						

### 3.7 Sensitivity analysis

Sensitivity analyses were performed for results with significant heterogeneity (Supplementary Figure S32–S38). Sensitivity analyses indicated that the results were stable regarding hospital length of stay, operative time, stay of postoperative and deep venous thrombosis, while the results were not stable regarding estimated blood loss and patients requiring ICU stay.

## 4 Discussion

### 4.1 A general interpretation of the results in the context of other evidence

This updated meta-analysis was conducted to compare the safety and efficacy of PEVAR with CEVAR in the treatment of TBAD. This meta-analysis demonstrated that PEVAR, in comparison to CEVAR, was associated with reduced hospital length of stay, decreased operative time, shorter postoperative duration, lower rates of groin infection, fewer heart-related complications, and a diminished incidence of lymphocele; however, it exhibited a higher occurrence of pseudoaneurysm and an increased rate of surgical suture failure. Besides, there was no significant difference between the two groups regarding estimated blood loss, patients requiring ICU stay, hematoma, acute kidney injury, lower extremity revascularization, ischemic colitis and deep venous thrombosis.

PEVAR was associated with reduced hospital stays, surgical durations, and postoperative recovery intervals, likely due to the smaller incision and the simplicity of executing PEVAR (40). The PEVAR group exhibited a reduced incidence of groin infections, lymphoceles, and cardiac complications. These enhanced outcomes to improvements in user experience and comfort with percutaneous access. Both open and percutaneous access methods entail hazards of infection. The open surgical method necessitates a wider incision, which may increase the risk of surgical site infections, and the carriage of Staphylococcus aureus may be significant (41). The percutaneous technique enables the execution of the same complex surgery in a far less invasive manner, resulting in less surgical site problems and utilizing local anesthesia. This may be highly beneficial in urgent situations where hemodynamic instability prevents the administration of general anesthesia (10). Furthermore, open femoral access has demonstrated the ability to generate substantial scar tissue, which may restrict future groin access for subsequent operations related to endoleak management or further graft maintenance (42).

The findings indicated that PEARV had certain drawbacks in comparison to CEVAR, primarily evidenced by an increased occurrence of surgical suture failure and pseudoaneurysm. Percutaneous access facilitates a smaller incision; nonetheless, the absence of control may result in access-related vascular injury, including stenosis or pseudoaneurysm. The device's failure to convert may result in increased blood loss and necessitate emergency surgery (42). In this meta-analysis, the success rate in the PEVAR group was approximately 95.0%. While it was 97.7% in the CEVAR group. This may be attributed to PEVAR's dependence on experience for identifying the puncture site, while CEVAR can be executed with direct visualization. Surgeons must complete training and practice to competently perform surgical procedures; hence, the risk of arterial puncture or vascular suture failures may increase during the early phases of this technology's use (33). The sheath's dimensions substantially affect the surgical procedure's success percentage. Previous research indicated that bigger sheath sizes correlated with an increased chance of closure failure, and a higher ratio of sheath size to CFA diameter was associated with an elevated risk of failure (43). Although the success rate of PEVAR is slightly lower than that of CEVAR, the safety profile of PEVAR is deemed acceptable, as unsuccessful cases can be promptly transitioned to CEVAR. It is essential to acknowledge that multiple factors can affect the success rate. Ultrasound guiding has been proposed to correlate with technical success in PEVAR (26). While there are no absolute contraindications for PEVAR, numerous studies have highlighted characteristics that may lead to procedural failure and need conversion to open femoral access. Consistent anatomic variables linked to PEVAR failure include femoral artery calcification exceeding 50% of vessel circumference and a femoral artery diameter of less than 5 mm (44, 45). Open femoral exposure should be contemplated in patients exhibiting these anatomical characteristics. Obesity and arterial calcification can influence the success rate; prior studies have indicated that obesity and significant arterial calcification are primary causes of hemostasis failure (45).

### 4.2 Any limitations of the evidence included in the review

First, the majority of the included studies were retrospective or prospective cohort studies, with a very small number of randomized controlled trials. This constrained the overall quality and robustness of the outcomes. Second, the funnel plots for surgical suture failure, hematoma and lymphocele indicated that the studies included in the analysis had significant asymmetry, which may result in an exaggeration of statistical significance (e.g., *P* values), heighten the likelihood of Type I error (false positive), and induce heterogeneity. Third, significant discrepancies in various factors across studies, such as patient baseline data, surgical techniques, urgical proficiency and equipment may potentially result in notable heterogeneity and compromise the validity of pooled outcome analyses. Besides, the included studies were marked by relatively brief follow-up durations, predominantly confined to a 30-day observation period, with just a minor fraction extending beyond one year. This time constraint may potentially influence the generalizability of our findings about long-term effects.

### 4.3 Any limitations of the review processes used

The literature search strategy, while thorough across PubMed, Embase, Web of Science, and Cochrane Library, may not have identified all pertinent studies due to discrepancies in database indexing or unpublished data, hence introducing potential selection bias. Moreover, statistical heterogeneity was pronounced for several aggregated outcomes, potentially because to substantial variations in multiple parameters among studies, including patient baseline characteristics, surgical methodologies, surgical expertise, and instrumentation. However, we were unable to conduct subgroup or meta-regression studies to investigate its sources due to our inadequate statistical proficiency and the lack of accurate outcome data. Besides, we failed to perform multivariate analysis or forest blot due to our inadequate statistical proficiency.

### 4.4 Implications of the results for practice, policy, and future research

The increasing utilization of percutaneous endovascular aneurysm repair (PEVAR) signifies its revolutionary impact in vascular surgery. In contrast to the previous meta-analyses (13, 14), this meta-analysis included more studies published in recent years and finally incorporated a total of 24 high-quality studies, with more outcomes and more deep search, hence yielding more robust and reliable conclusions. This meta-analysis indicated that PEVAR, relative to CEVAR, was linked to a reduced hospital length of stay, decreased operational duration, shorter postoperative recovery, lower rates of groin infection, fewer cardiac problems, and a reduced incidence of lymphocele. These benefits correspond with contemporary value-based care models that prioritize minimally invasive methods and expedited recovery processes. Practitioners must weigh these advantages against CEVAR's reduced incidence of suture failure and pseudoaneurysm development, especially in patients with significant femoral calcification or obesity. The technical success rate of PEVAR highlights the significance of operator proficiency and patient selection criteria, including a femoral artery diameter exceeding 5 mm and low circumferential calcification. Future research should concentrate on enhancing the success rate of PEVAR surgery and mitigating the occurrence of pseudoaneurysm, including advancements in PEVAR equipment and improvement of doctors' surgical abilities.

In conclusion, relative to CEVAR, PEVAR was linked to a shorter hospital stay, reduced operative duration, briefer postoperative recovery, lower groin infection rates, fewer cardiac complications, and a decreased incidence of lymphocele; nevertheless, it demonstrated a higher prevalence of pseudoaneurysm and an elevated rate of surgical suture failure. PEVAR was a secure and efficacious approach for the management of TBAD.

## Data Availability

The datasets presented in this study can be found in online repositories. The names of the repository/repositories and accession number(s) can be found in the article/Supplementary Material.

## Supplementary material

Supplementary Data Sheet 1 Comprehensive listing of the search results.

Supplementary Data Sheet 2 Supplement Figures.
