# Supplementary material for: Comparison of percutaneous vs. cutdown access for endovascular aortic repair in the treatment of type B aortic dissection: a meta-analysis
Source: Front Cardiovasc Med. 2025 Nov 24;12:1673817. doi: 10.3389/fcvm.2025.1673817 (PMC12682885; doi:10.3389/fcvm.2025.1673817)
Supplement: Supplementary Data Sheet 1 — Comprehensive listing of the search results. [file Datasheet1.pdf]

PubMed		
No.	Query	Results
1	(((percutaneous[Title/Abstract]) OR (PEVAR[Title/Abstract])) OR (PeRcutanEous[Title/Abstract])) OR (Closure[Title/Abstract])	240,069
2	((((open[Title/Abstract]) OR (cutdown[Title/Abstract])) OR (exposure[Title/Abstract])) OR (SEVAR[Title/Abstract])) OR (surgical[Title/Abstract])	2,214,561
3	((aortic aneurysm repair[Title/Abstract]) OR (Aneurysms, Aortic[Title/Abstract])) OR (Aneurysm, Aortic[Title/Abstract])	4,910
4	(((((((type B aortic dissection[Title/Abstract]) OR (Abdominal Aortic Aneurysms[Title/Abstract])) OR (Aneurysm, Abdominal Aorta[Title/Abstract])) OR (Abdominal Aortic Aneurysm[Title/Abstract])) OR (Aneurysm, Abdominal Aortic[Title/Abstract])) OR (Abdominal Aorta Aneurysm[Title/Abstract])) OR (Abdominal Aorta Aneurysms[Title/Abstract])) OR (Aorta Aneurysm, Abdominal[Title/Abstract])	20589
5	#1 AND #2 AND #3 AND #4	119

Web of science		
No.	Query	Results
1	TS=(percutaneous OR PEVAR OR PeRcutanEous OR Closure)	700823
2	TS=(open OR cutdown OR exposure OR SEVAR OR surgical)	12569777
3	TS=(aortic aneurysm repair OR Aneurysms, Aortic OR Aneurysm, Aortic)	81076
4	TS=(type B aortic dissection OR Abdominal Aortic Aneurysms OR Aneurysm, Abdominal Aorta OR Abdominal Aortic Aneurysm OR Aneurysm, Abdominal Aortic OR Abdominal Aorta Aneurysm OR Abdominal Aorta Aneurysms OR Aorta Aneurysm, Abdominal)	44539
	#1 AND #2 AND #3 AND #4	772

Embase		
No.	Query	Results
1	('open' OR 'cutdown' OR 'exposure' OR 'sevar' OR 'surgical') AND [2000-2024]/py	4304597
2	('aortic aneurysm repair' OR 'aneurysms, aortic' OR 'aneurysm,	6776

	aortic') AND [2000-2024]/py	
3	('pevar' OR 'percutaneous' OR 'closure') AND [2000-2024]/py	477922
4	'type b aortic dissection'/exp OR 'debakey type iii aortic dissection' OR 'descending aorta dissection' OR 'descending aortic dissection' OR 'descending thoracic aorta dissection' OR 'descending thoracic aortic dissection' OR 'dissection of descending aorta' OR 'dissection of the descending thoracic aorta' OR 'distal aortic dissection' OR 'stanford type b aortic dissection' OR 'type b stanford aortic dissection' OR 'type iii aortic dissection' OR 'type b aortic dissection' OR 'abdominal aortic aneurysms' OR 'aneurysm, abdominal aorta' OR 'abdominal aortic aneurysm' OR 'aneurysm, abdominal aortic' OR 'abdominal aorta aneurysm' OR 'abdominal aorta aneurysms' OR 'aorta aneurysm, abdominal'	44234
5	#1 AND #2 AND #3 AND #4	277

cochrane library		
No.	Query	Results
1	percutaneous OR PEVAR OR PeRcutanEous OR Closure	42714
2	open OR cutdown OR exposure OR SEVAR OR surgical	362180
3	aortic aneurysm repair OR Aneurysms, Aortic OR Aneurysm, Aortic	2454
4	Aneurysm, Abdominal Aorta OR Abdominal Aortic Aneurysm OR Aneurysm, Abdominal Aortic OR Abdominal Aorta Aneurysm OR Abdominal Aorta Aneurysms OR Aorta Aneurysm, Abdominal OR type B aortic dissection	1967
5	#1 AND #2 AND #3 AND #4	34
