# Supplementary material for: Comparison of percutaneous vs. cutdown access for endovascular aortic repair in the treatment of type B aortic dissection: a meta-analysis
Source: Front Cardiovasc Med. 2025 Nov 24;12:1673817. doi: 10.3389/fcvm.2025.1673817 (PMC12682885; doi:10.3389/fcvm.2025.1673817)
Supplement: Supplementary Data Sheet 2 — Supplement Figures. [file Datasheet2.pdf]

Hospital length of stay

Figure1 **Forest plot of the meta-analysis for Hospital length of stay**

Study or Subgroup	pEVAR Mean	pEVAR SD	pEVAR Total	cEVAR Mean	cEVAR SD	cEVAR Total	Weight	Mean Difference IV, Random, 95% CI
Akbulut 2022	2.5	1.7	65	4.7	2.5	87	13.9%	-2.20 [-2.87, -1.53]
Altoijry 2023	11.6	12.09	35	21.54	21.49	24	0.8%	-9.94 [-19.42, -0.46]
Baxter 2021	4.06	5.89	2017	6.15	7.6	2445	14.6%	-2.09 [-2.49, -1.69]
Hahl 2023	3	1.9	257	3.5	1.7	186	14.7%	-0.50 [-0.84, -0.16]
Sahin 2020	9.09	4.74	56	10.06	4.96	40	8.6%	-0.97 [-2.95, 1.01]
Taku 2015	6.7	6.8	50	9.3	4.5	93	8.2%	-2.60 [-4.70, -0.50]
Tay 2022	11	2.8	21	16	1.1	14	11.3%	-5.00 [-6.33, -3.67]
Thurston 2019	1.3	1.18	50	2.03	1.38	50	14.3%	-0.73 [-1.23, -0.23]
Wu 2022	5.19	2.26	178	8.33	3.76	117	13.6%	-3.14 [-3.90, -2.38]
**Total (95% CI)**			**2729**			**3056**	**100.0%**	**-2.16 [-3.05, -1.27]**

Heterogeneity: Tau² = 1.37; Chi² = 104.20, df = 8 (P < 0.00001); I² = 92%
Test for overall effect: Z = 4.75 (P < 0.00001)

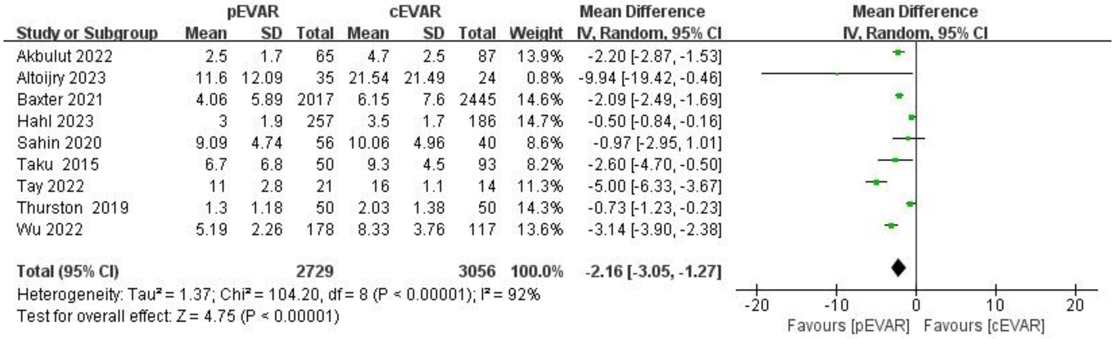

Operative time

Figure2 Forest plot of the meta-analysis for Operative time

Study or Subgroup	pEVAR Mean	pEVAR SD	pEVAR Total	cEVAR Mean	cEVAR SD	cEVAR Total	Weight	Mean Difference IV, Random, 95% CI
Akbulut 2022	102.5	30.9	65	126.3	44.9	87	9.5%	-23.80 [-35.86, -11.74]
Howell 2002	105	21	30	171	33	96	10.1%	-66.00 [-76.00, -56.00]
Kauvar 2016	134	65	1533	150	69	1589	11.2%	-16.00 [-20.70, -11.30]
Lin 2018	94.17	23.24	60	120.36	26.31	55	10.3%	-26.19 [-35.30, -17.08]
Nelson 2014	100.9	40.5	101	141	73	50	6.8%	-40.10 [-61.82, -18.38]
Sahin 2020	104.3	13.8	56	160	25.4	40	10.4%	-55.70 [-64.36, -47.04]
Siracuse 2018	114.5	58.4	8340	147.1	71.5	4747	11.5%	-32.60 [-34.99, -30.21]
Taku 2015	153	47	50	211	88	96	6.8%	-58.00 [-79.90, -36.10]
Tay 2022	136	61	21	260	13	14	5.6%	-124.00 [-150.96, -97.04]
Torsello 2003	86.7	27	15	107.8	38.5	15	6.3%	-21.10 [-44.90, 2.70]
Wu 2022	46	18.52	178	72	7.78	117	11.4%	-26.00 [-29.06, -22.94]
**Total (95% CI)**			**10449**			**6906**	**100.0%**	**-40.87 [-49.72, -32.02]**

Heterogeneity: Tau² = 175.78; Chi² = 184.35, df = 10 (P < 0.00001); I² = 95%
Test for overall effect: Z = 9.06 (P < 0.00001)

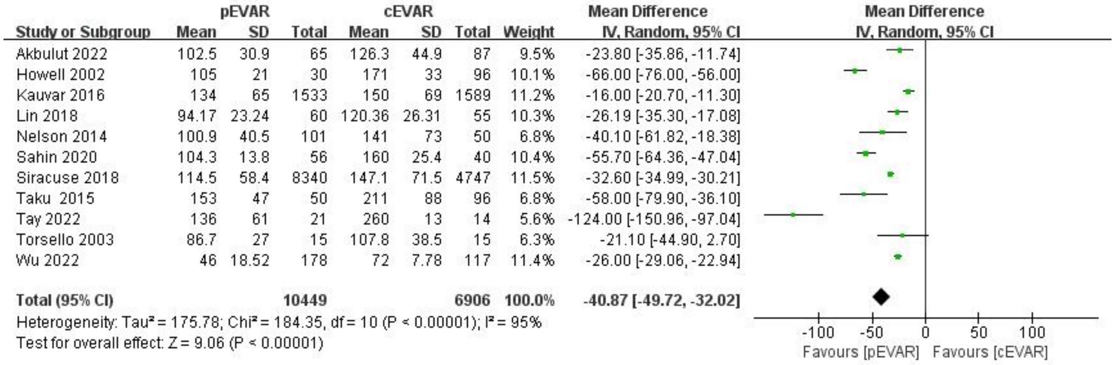

Estimated blood loss

Figure3 Forest plot of the meta-analysis for Estimated blood loss

Study or Subgroup	pEVAR Mean	pEVAR SD	pEVAR Total	cEVAR Mean	cEVAR SD	cEVAR Total	Weight	Mean Difference IV, Random, 95% CI
Christopher 2017	170	16	102	480	35.9	98	17.0%	-310.00 [-317.76, -302.24]
Howell 2002	90.6	50	30	383	410	96	16.1%	-292.40 [-376.34, -208.46]
Lin 2018	126.55	61.83	60	175.45	77.33	55	16.9%	-48.90 [-74.64, -23.16]
Nelson 2014	202.9	200.7	101	280	290	50	16.0%	-77.10 [-166.51, 12.31]
Siracuse 2018	248.8	489.3	8340	276.5	627	4747	16.9%	-27.70 [-48.40, -7.00]
Wu 2022	25	7.4	178	45	7.4	117	17.0%	-20.00 [-21.73, -18.27]
**Total (95% CI)**			**8811**			**5163**	**100.0%**	**-128.55 [-277.51, 20.40]**

Heterogeneity: Tau² = 33976.87; Chi² = 5155.66, df = 5 (P < 0.00001); I² = 100%
Test for overall effect: Z = 1.69 (P = 0.09)

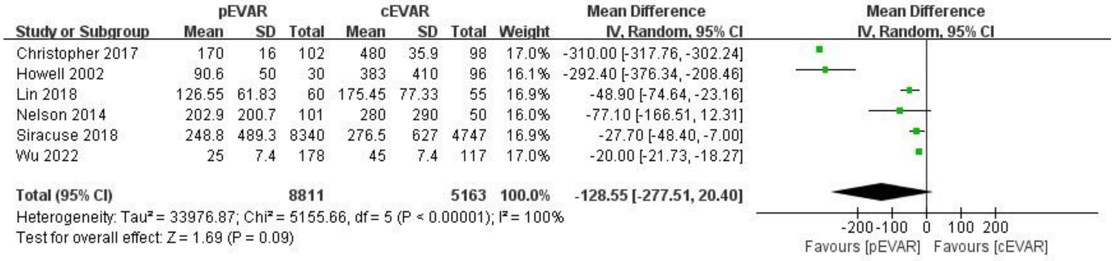

Stay of postoperative

Figure4 **Forest plot of the meta-analysis for Stay of postoperative**

Study or Subgroup	pEVAR Mean	pEVAR SD	pEVAR Total	cEVAR Mean	cEVAR SD	cEVAR Total	Weight	Mean Difference IV, Random, 95% CI
Christopher 2017	1.7	0.19	102	3	0.58	98	26.6%	-1.30 [-1.42, -1.18]
Kauvar 2016	2.3	3.1	1533	2.5	3.5	1589	25.8%	-0.20 [-0.43, 0.03]
Lin 2018	5.12	1.35	60	6.85	1.55	55	21.7%	-1.73 [-2.26, -1.20]
Siracuse 2018	2.3	5.3	8340	3.2	6.4	4747	25.9%	-0.90 [-1.11, -0.69]
**Total (95% CI)**			**10035**			**6489**	**100.0%**	**-1.01 [-1.56, -0.45]**

Heterogeneity: Tau² = 0.30; Chi² = 76.31, df = 3 (P < 0.00001); I² = 96%
Test for overall effect: Z = 3.53 (P = 0.0004)

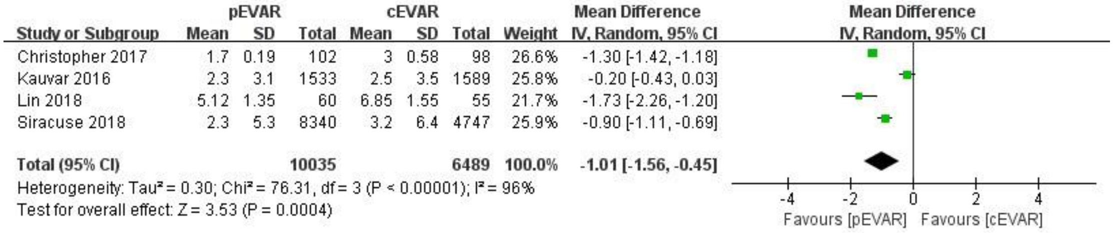

Patients requiring ICU stay

Figure5 Forest plot of the meta-analysis for Patients requiring ICU stay

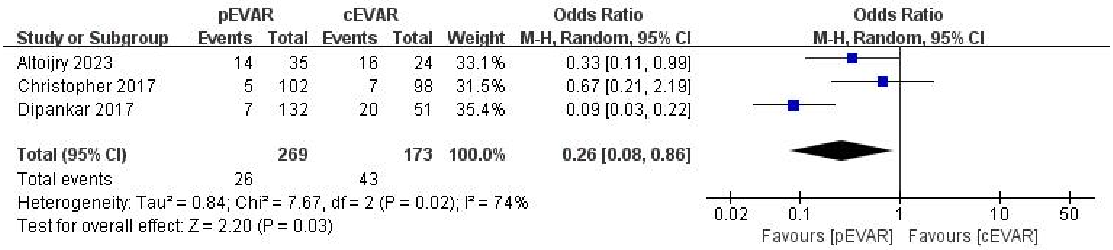

Surgical suture failure

Figure6 Forest plot of the meta-analysis for Surgical suture failure

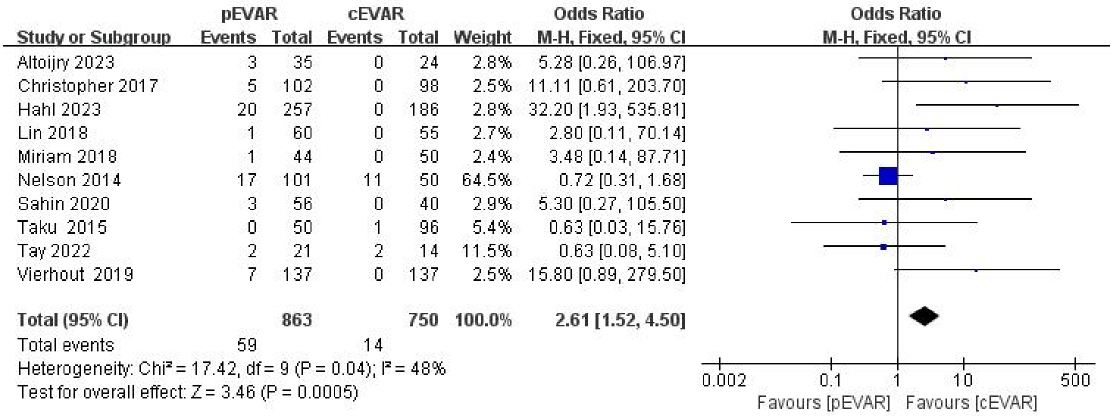

Pseudoaneurysm

Figure7 Forest plot of the meta-analysis for Pseudoaneurysm

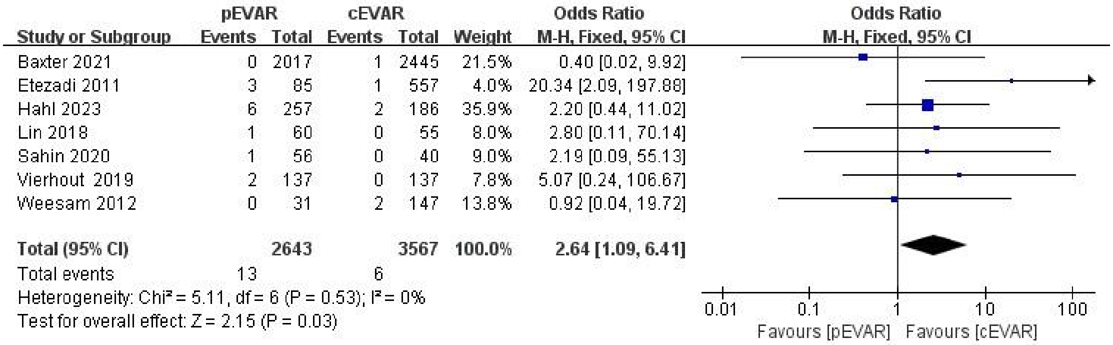

Hematoma

Figure8 Forest plot of the meta-analysis for Hematoma

Study or Subgroup	pEVAR Events	pEVAR Total	cEVAR Events	cEVAR Total	Weight	Odds Ratio M-H, Fixed, 95% CI
Altoijry 2023	5	35	3	24	8.5%	1.17 [0.25, 5.42]
Baxter 2021	1	2017	1	2445	2.5%	1.21 [0.08, 19.39]
Christopher 2017	10	102	2	98	5.1%	5.22 [1.11, 24.46]
Etezadi 2011	2	85	34	557	24.4%	0.37 [0.09, 1.57]
Lin 2018	0	60	2	55	7.2%	0.18 [0.01, 3.77]
Miriam 2018	0	44	2	50	6.4%	0.22 [0.01, 4.67]
Sahin 2020	2	56	3	40	9.4%	0.46 [0.07, 2.87]
Smith 2009	1	38	5	50	11.7%	0.24 [0.03, 2.17]
Tay 2022	1	21	1	14	3.2%	0.65 [0.04, 11.33]
Vierhout 2019	5	137	4	137	10.7%	1.26 [0.33, 4.79]
Weesam 2012	1	31	8	147	7.5%	0.58 [0.07, 4.81]
Wu 2022	1	178	1	117	3.3%	0.66 [0.04, 10.58]
**Total (95% CI)**		**2804**		**3734**	**100.0%**	**0.81 [0.49, 1.32]**
Total events	29		66			

Heterogeneity: Chi² = 10.76, df = 11 (P = 0.46); I² = 0%
Test for overall effect: Z = 0.86 (P = 0.39)

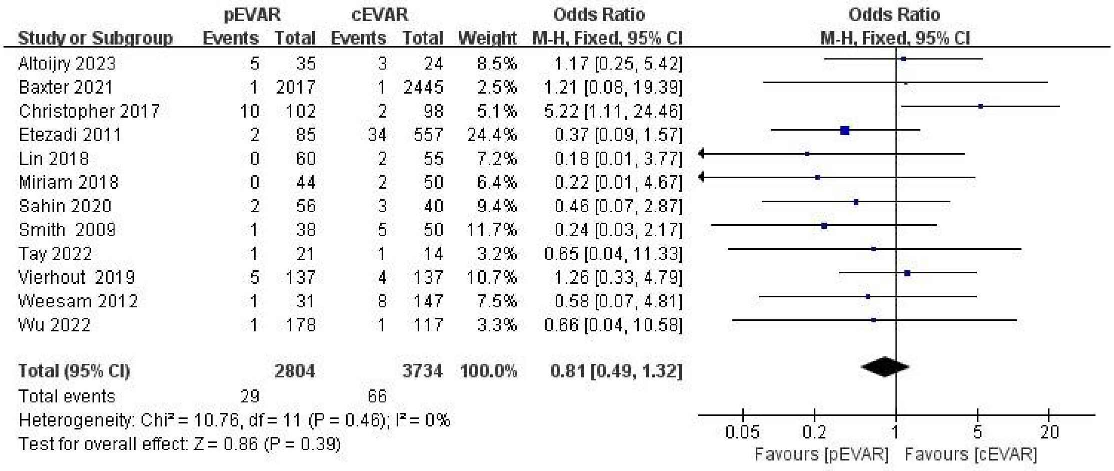

Groin infection

Figure9 Forest plot of the meta-analysis for Groin infection

Study or Subgroup	pEVAR Events	pEVAR Total	cEVAR Events	cEVAR Total	Weight	Odds Ratio M-H, Fixed, 95% CI
Akbulut 2022	2	65	14	87	13.1%	0.17 [0.04, 0.76]
Altoijry 2023	7	35	3	24	3.2%	1.75 [0.40, 7.58]
Baxter 2021	1	2017	1	2445	1.0%	1.21 [0.08, 19.39]
Christopher 2017	0	102	2	98	2.9%	0.19 [0.01, 3.97]
Dominique 2015	11	1108	60	3004	36.2%	0.49 [0.26, 0.94]
Etezadi 2011	1	85	3	557	0.9%	2.20 [0.23, 21.38]
Hahl 2023	1	257	5	186	6.5%	0.14 [0.02, 1.22]
Lin 2018	0	65	2	55	3.0%	0.16 [0.01, 3.48]
Miriam 2018	0	44	10	50	11.0%	0.04 [0.00, 0.76]
Peter 2020	0	49	1	24	2.2%	0.16 [0.01, 4.03]
Sahin 2020	0	56	1	40	2.0%	0.23 [0.01, 5.87]
Siracuse 2018	7	8340	5	4747	7.2%	0.80 [0.25, 2.51]
Smith 2009	0	38	2	50	2.4%	0.25 [0.01, 5.40]
Taku 2015	0	50	1	96	1.2%	0.63 [0.03, 15.76]
Tay 2022	3	21	0	14	0.6%	5.49 [0.26, 114.91]
Vierhout 2019	0	137	3	137	3.9%	0.14 [0.01, 2.73]
Weesam 2012	0	31	1	147	0.6%	1.55 [0.06, 38.94]
Wu 2022	0	178	1	117	2.0%	0.22 [0.01, 5.39]
**Total (95% CI)**		**12678**		**11878**	**100.0%**	**0.44 [0.30, 0.65]**
Total events	33		115			

Heterogeneity: Chi² = 17.52, df = 17 (P = 0.42); I² = 3%
Test for overall effect: Z = 4.15 (P < 0.0001)

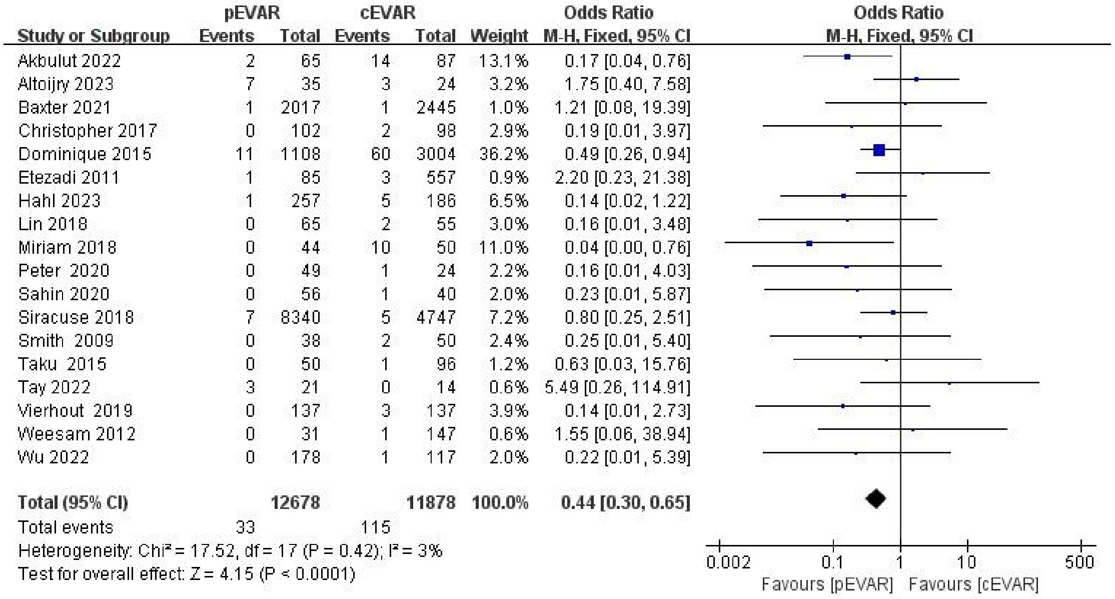

Heart-related complications

Figure10 Forest plot of the meta-analysis for Heart-related complications

Study or Subgroup	pEVAR Events	pEVAR Total	cEVAR Events	cEVAR Total	Weight	Odds Ratio M-H, Fixed, 95% CI
Christopher 2017	0	102	2	98	2.0%	0.19 [0.01, 3.97]
Dipankar 2017	2	132	1	51	1.1%	0.77 [0.07, 8.67]
Dominique 2015	8	1108	39	3004	16.6%	0.55 [0.26, 1.19]
Kauvar 2016	15	1533	18	1589	14.0%	0.86 [0.43, 1.72]
Peter 2020	5	49	1	24	1.0%	2.61 [0.29, 23.72]
Siracuse 2018	88	8340	64	4747	64.4%	0.78 [0.56, 1.08]
Tay 2022	2	21	1	14	0.9%	1.37 [0.11, 16.70]
**Total (95% CI)**		**11285**		**9527**	**100.0%**	**0.76 [0.59, 1.00]**
Total events	120		126			

Heterogeneity: Chi² = 3.04, df = 6 (P = 0.80); I² = 0%
Test for overall effect: Z = 1.99 (P = 0.05)

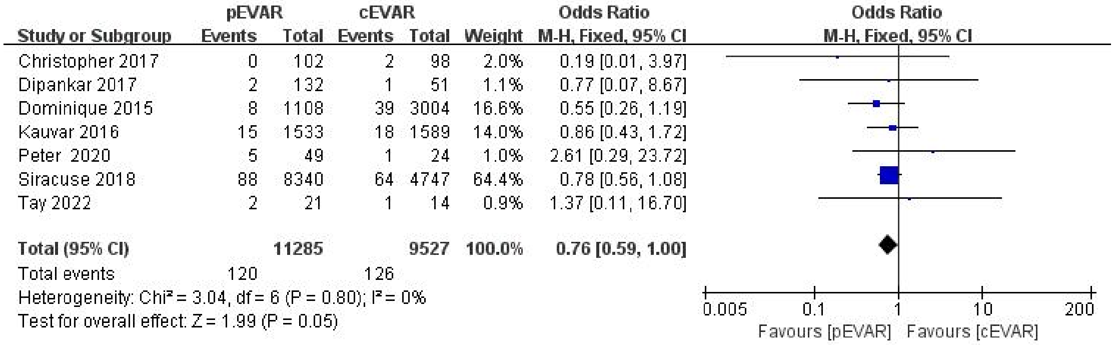

Lymphocele

Figure11 Forest plot of the meta-analysis for Lymphocele

Study or Subgroup	pEVAR Events	pEVAR Total	cEVAR Events	cEVAR Total	Weight	Odds Ratio M-H, Fixed, 95% CI
Akbulut 2022	0	65	6	87	23.2%	0.10 [0.01, 1.73]
Lin 2018	0	60	2	55	10.9%	0.18 [0.01, 3.77]
Nelson 2014	0	101	1	50	8.4%	0.16 [0.01, 4.06]
Peter 2020	17	49	5	24	18.4%	2.02 [0.64, 6.36]
Taku 2015	0	50	2	96	7.2%	0.37 [0.02, 7.95]
Torsello 2003	0	15	3	15	14.3%	0.12 [0.01, 2.45]
Wu 2022	0	178	3	117	17.7%	0.09 [0.00, 1.79]
**Total (95% CI)**		**518**		**444**	**100.0%**	**0.49 [0.24, 0.98]**
Total events	17		22			

Heterogeneity: Chi² = 10.08, df = 6 (P = 0.12); I² = 40%
Test for overall effect: Z = 2.03 (P = 0.04)

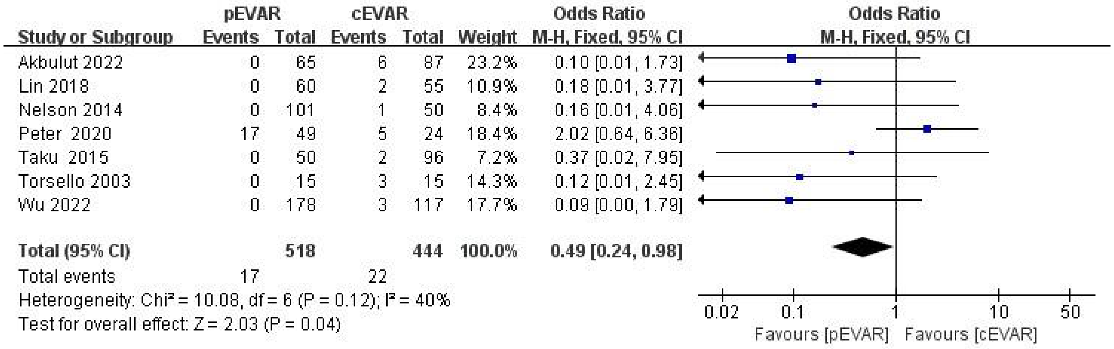

Acute kidney injury

Figure12 Forest plot of the meta-analysis for Acute kidney injury

Study or Subgroup	pEVAR Events	pEVAR Total	cEVAR Events	cEVAR Total	Weight	Odds Ratio M-H, Fixed, 95% CI
Dipankar 2017	2	132	1	51	4.5%	0.77 [0.07, 8.67]
Dominique 2015	4	1108	15	3004	25.5%	0.72 [0.24, 2.18]
Kauvar 2016	11	1533	17	1589	52.5%	0.67 [0.31, 1.43]
Nelson 2014	3	101	1	50	4.1%	1.50 [0.15, 14.80]
Peter 2020	10	49	3	24	10.1%	1.79 [0.44, 7.24]
Tay 2022	3	21	1	14	3.3%	2.17 [0.20, 23.25]
**Total (95% CI)**		**2944**		**4732**	**100.0%**	**0.88 [0.53, 1.47]**
Total events	33		38			

Heterogeneity: Chi² = 2.40, df = 5 (P = 0.79); I² = 0%
Test for overall effect: Z = 0.47 (P = 0.64)

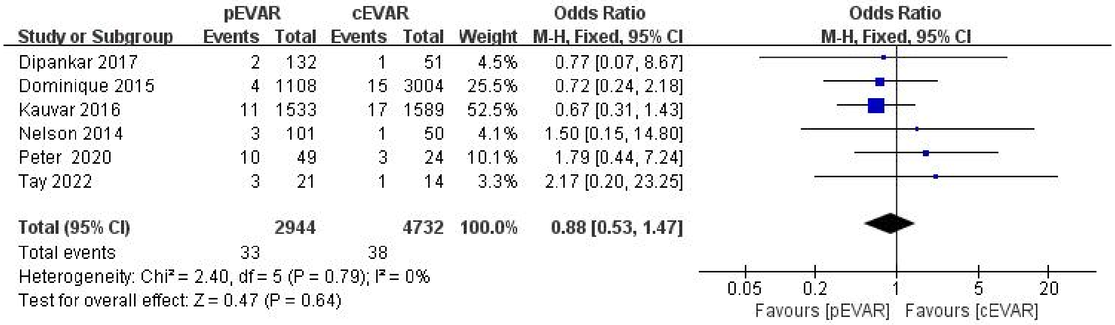

Lower extremity revascularization

Figure13 Forest plot of the meta-analysis for Lower extremityrevascularization

Study or Subgroup	pEVAR Events	pEVAR Total	cEVAR Events	cEVAR Total	Weight	Odds Ratio M-H, Fixed, 95% CI
Akbulut 2022	3	65	3	87	3.2%	1.35 [0.26, 6.94]
Baxter 2021	0	2017	3	2445	4.1%	0.17 [0.01, 3.35]
Christopher 2017	2	102	1	98	1.3%	1.94 [0.17, 21.74]
Dipankar 2017	1	132	1	51	1.9%	0.38 [0.02, 6.22]
Dominique 2015	18	1108	36	3004	25.0%	1.36 [0.77, 2.41]
Lin 2018	1	60	0	55	0.7%	2.80 [0.11, 70.14]
Miriam 2018	0	44	1	50	1.8%	0.37 [0.01, 9.34]
Nelson 2014	3	101	2	50	3.4%	0.73 [0.12, 4.54]
Peter 2020	2	49	1	24	1.7%	0.98 [0.08, 11.36]
Siracuse 2018	71	8340	32	4747	53.0%	1.27 [0.83, 1.92]
Tay 2022	0	21	2	14	3.8%	0.12 [0.01, 2.62]
**Total (95% CI)**		**12039**		**10625**	**100.0%**	**1.17 [0.86, 1.58]**
Total events	101		82			

Heterogeneity: Chi² = 5.98, df = 10 (P = 0.82); I² = 0%
Test for overall effect: Z = 0.98 (P = 0.33)

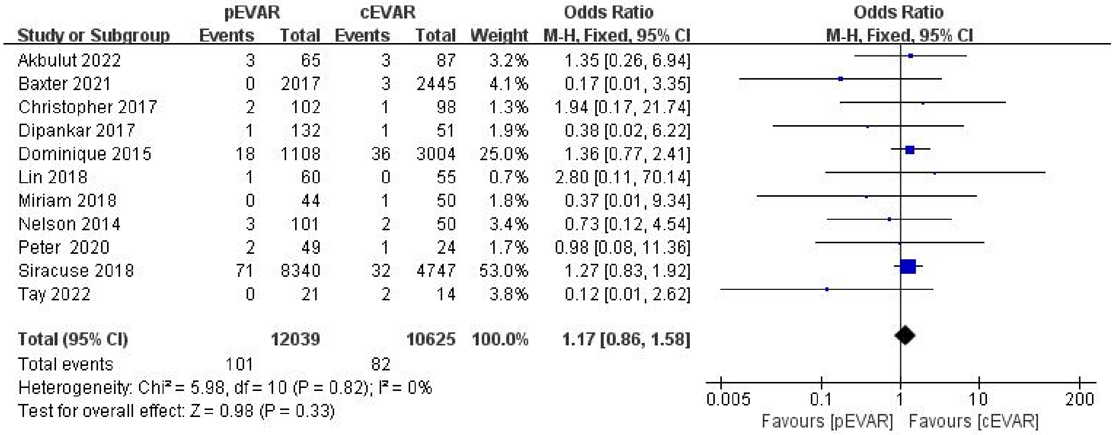

Ischemic colitis

Figure14 Forest plot of the meta-analysis for Ischemic colitis

Study or Subgroup	pEVAR Events	pEVAR Total	cEVAR Events	cEVAR Total	Weight	Odds Ratio M-H, Fixed, 95% CI
Dominique 2015	6	1108	12	3004	67.3%	1.36 [0.51, 3.63]
Peter 2020	0	49	1	24	20.7%	0.16 [0.01, 4.03]
Tay 2022	1	21	1	14	12.0%	0.65 [0.04, 11.33]
**Total (95% CI)**		**1178**		**3042**	**100.0%**	**1.02 [0.42, 2.49]**
Total events	7		14			

Heterogeneity: Chi² = 1.69, df = 2 (P = 0.43); I² = 0%
Test for overall effect: Z = 0.05 (P = 0.96)

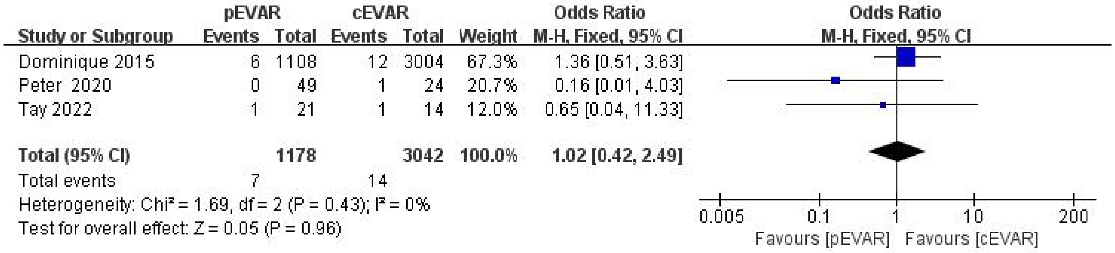

Deep venous thrombosis

Figure15 Forest plot of the meta-analysis for Deep venous thrombosis

Study or Subgroup	pEVAR Events	pEVAR Total	cEVAR Events	cEVAR Total	Weight	Odds Ratio M-H, Random, 95% CI
Dominique 2015	10	1108	12	3004	33.1%	2.27 [0.98, 5.27]
Etezadi 2011	1	85	1	557	12.9%	6.62 [0.41, 106.83]
Kauvar 2016	7	1533	16	1589	32.5%	0.45 [0.19, 1.10]
Lin 2018	1	60	0	55	10.5%	2.80 [0.11, 70.14]
Tay 2022	0	21	2	14	11.0%	0.12 [0.01, 2.62]
**Total (95% CI)**		**2807**		**5219**	**100.0%**	**1.14 [0.34, 3.84]**
Total events	19		31			

Heterogeneity: Tau² = 0.98; Chi² = 10.77, df = 4 (P = 0.03); I² = 63%
Test for overall effect: Z = 0.21 (P = 0.84)

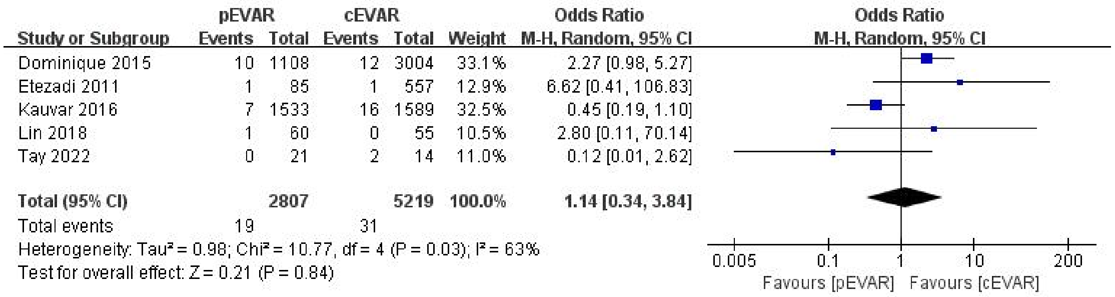

Figure16 Funnel plot for Hospital length of stay

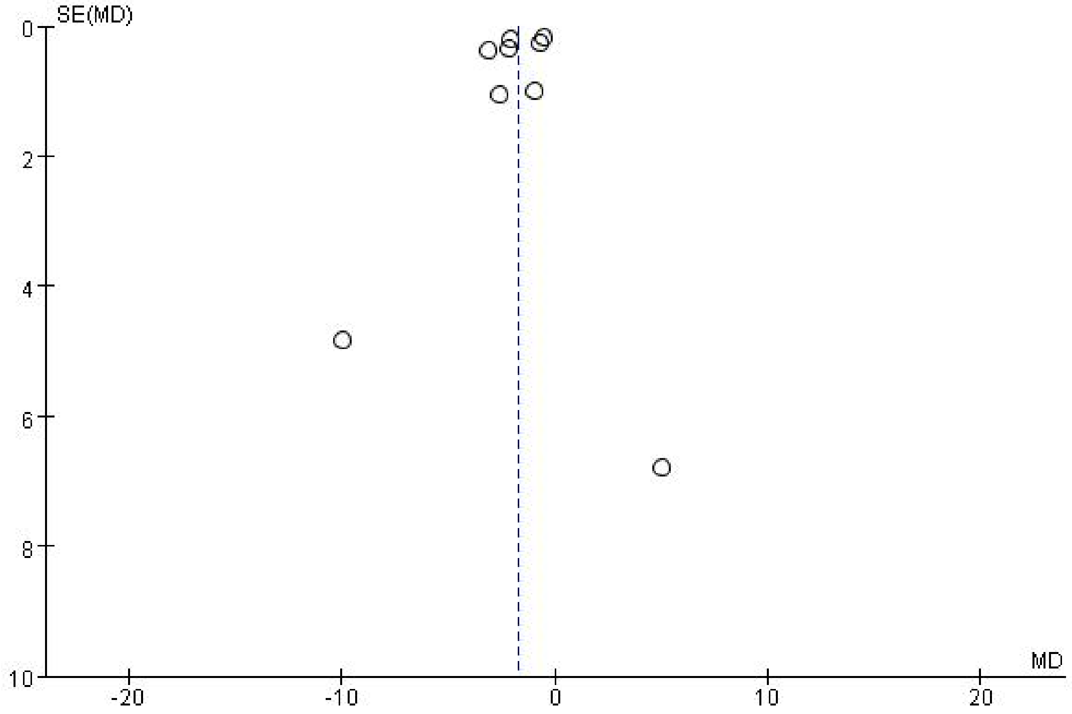

Figure17 **Funnel plot for Operative time**

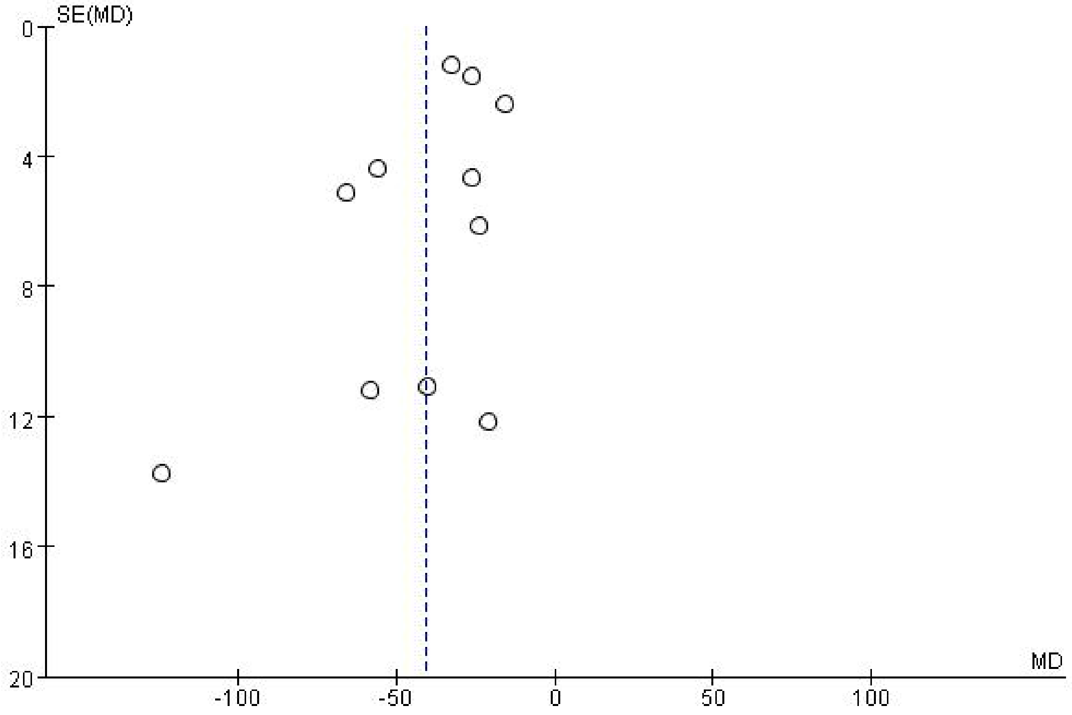

Figure18 **Funnel plot for Groin infection**

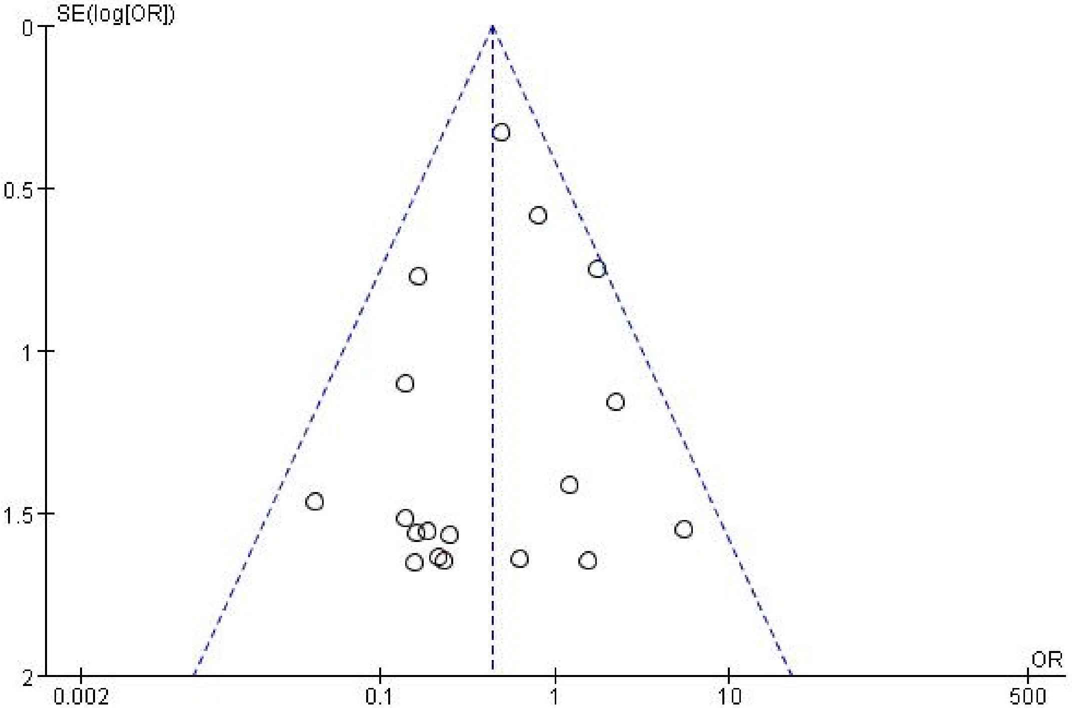

Figure19 **Funnel plot for Lower extremityrevascularization**

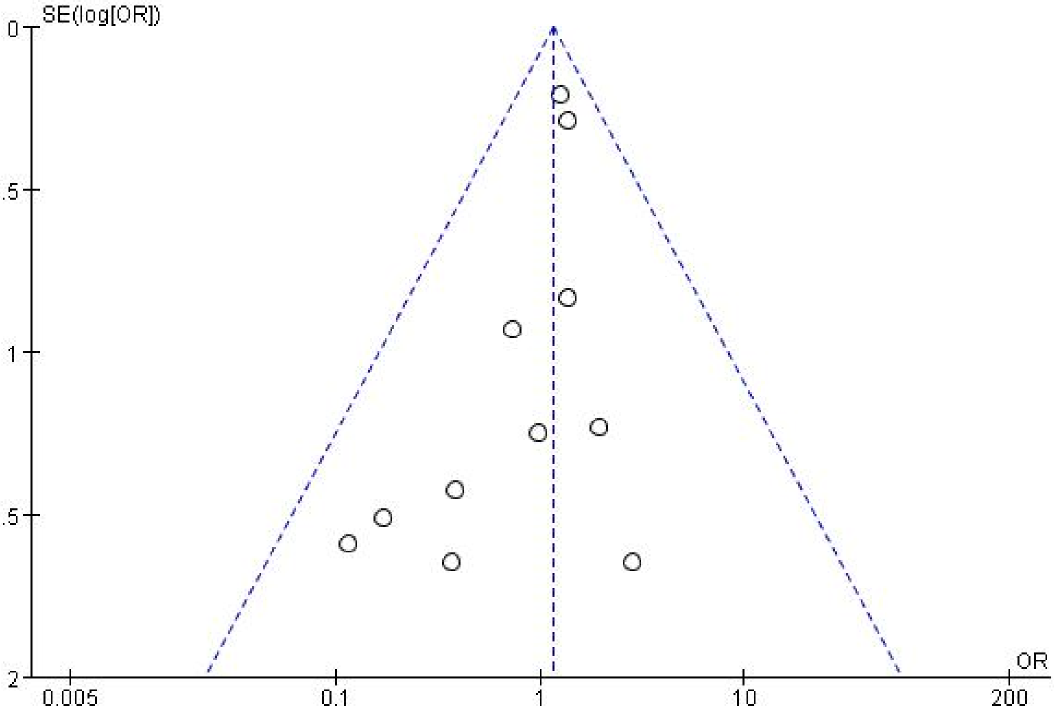

Figure20 Funnel plot for Surgical suture failure

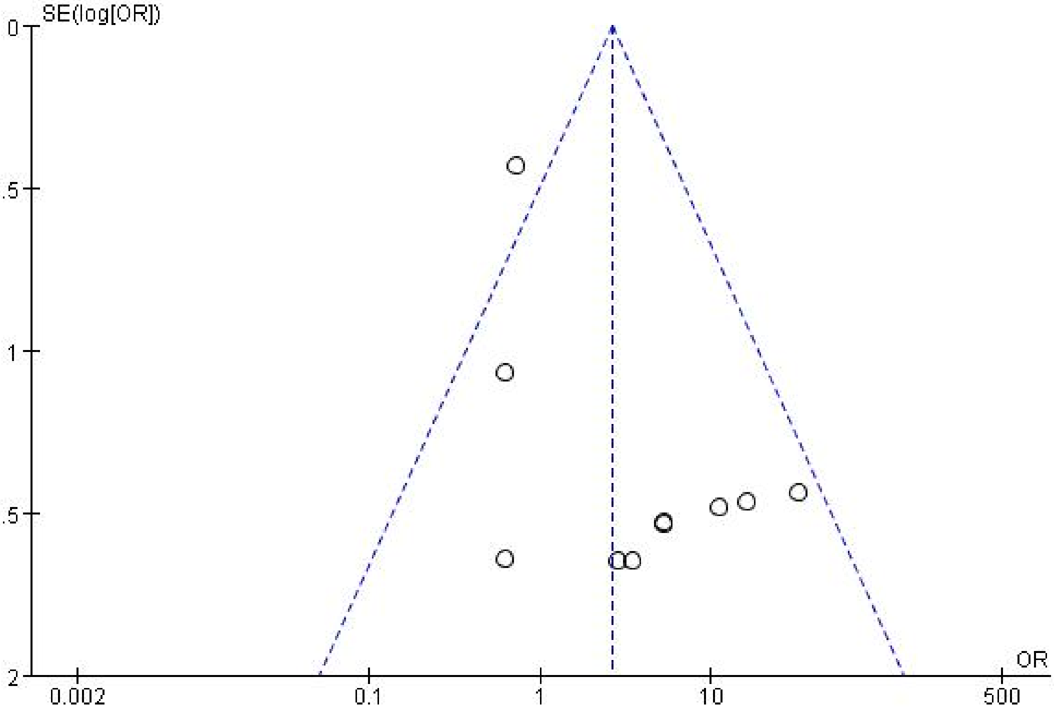

Figure21 Funnel plot for Hematoma

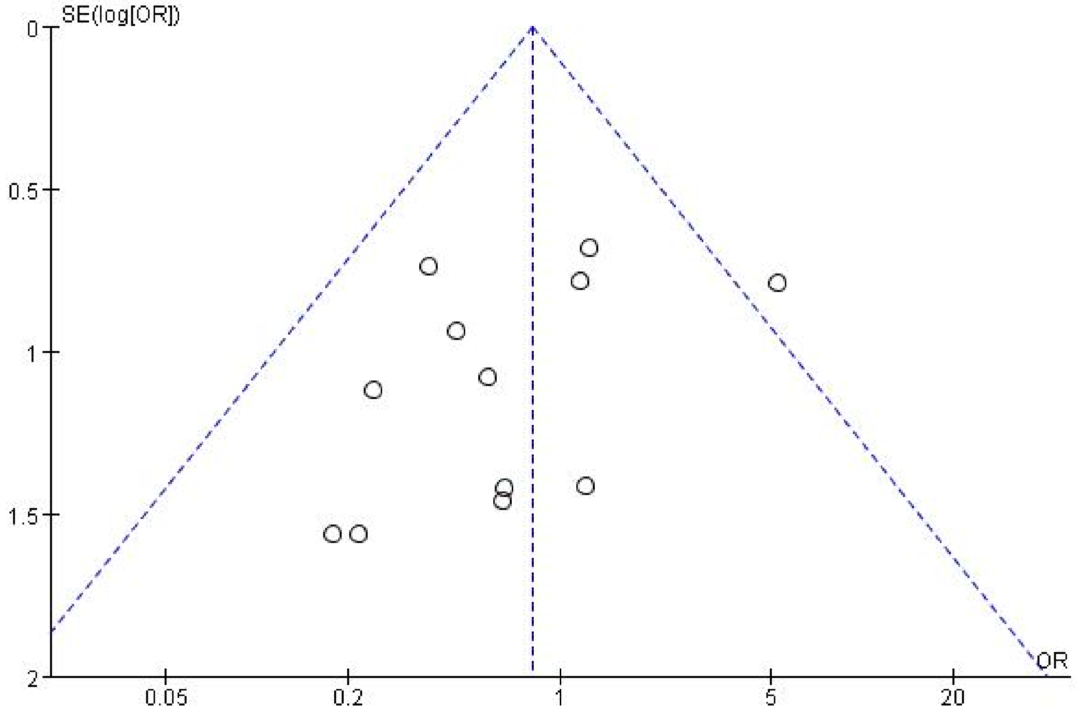

Figure22 Forest plot of literature on Hospital length of stay with a follow-up time of 6 months or more

	PEVAR			CEVAR				Mean Difference
Study or Subgroup	Mean	SD	Total	Mean	SD	Total	Weight	IV, Fixed, 95% CI
Akbulut 2022	2.5	1.7	65	4.7	2.5	87	12.4%	-2.20 [-2.87, -1.53]
Baxter 2021	4.06	5.89	2017	6.15	7.6	2445	35.5%	-2.09 [-2.49, -1.69]
Hahl 2023	3	1.9	257	3.5	1.7	186	48.9%	-0.50 [-0.84, -0.16]
Tay 2022	11	2.8	21	16	1.1	14	3.1%	-5.00 [-6.33, -3.67]
**Total (95% CI)**			**2360**			**2732**	**100.0%**	**-1.42 [-1.65, -1.18]**

Heterogeneity: Chi² = 72.71, df = 3 (P < 0.00001); I² = 96%
Test for overall effect: Z = 11.78 (P < 0.00001)

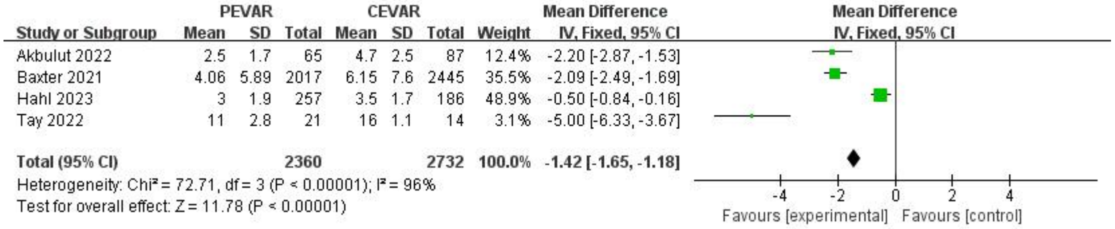

Figure23 Forest plot of literature on Operative time with a follow-up time of 6 months or more

	PEVAR			CEVAR				Mean Difference
Study or Subgroup	Mean	SD	Total	Mean	SD	Total	Weight	IV, Random, 95% CI
Akbulut 2022	102.5	30.9	65	126.3	44.9	87	34.5%	-23.80 [-35.86, -11.74]
Nelson 2014	100.9	40.5	101	141	73	50	33.2%	-40.10 [-61.82, -18.38]
Tay 2022	136	61	21	260	13	14	32.3%	-124.00 [-150.96, -97.04]
**Total (95% CI)**			**187**			**151**	**100.0%**	**-61.54 [-114.96, -8.11]**

Heterogeneity: Tau² = 2114.34; Chi² = 44.21, df = 2 (P < 0.00001); I² = 95%
Test for overall effect: Z = 2.26 (P = 0.02)

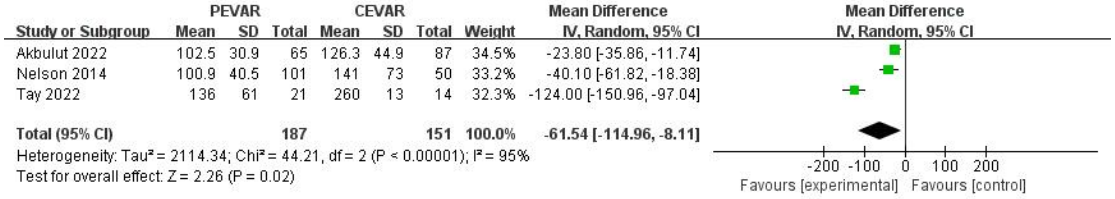

Figure24 Forest plot of literature on Surgical suture failure with a follow-up time of 6 months or more

	PEVAR		CEVAR			Odds Ratio
Study or Subgroup	Events	Total	Events	Total	Weight	M-H, Random, 95% CI
Hahl 2023	20	257	0	186	26.7%	32.20 [1.93, 535.81]
Nelson 2014	17	101	11	50	41.0%	0.72 [0.31, 1.68]
Tay 2022	2	21	2	14	32.3%	0.63 [0.08, 5.10]
**Total (95% CI)**		**379**		**250**	**100.0%**	**1.90 [0.18, 20.03]**
Total events	39		13			

Heterogeneity: Tau² = 3.33; Chi² = 9.67, df = 2 (P = 0.008); I² = 79%
Test for overall effect: Z = 0.54 (P = 0.59)

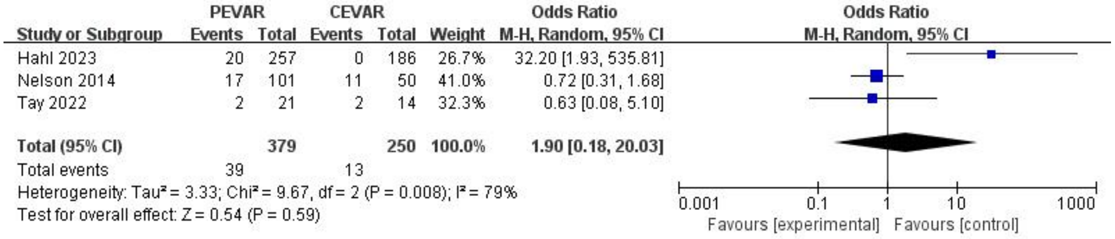

Figure25 Forest plot of literature on Hematomat with a follow-up time of 6 months or more

Study or Subgroup	PEVAR Events	PEVAR Total	CEVAR Events	CEVAR Total	Weight	Odds Ratio M-H, Fixed, 95% CI
Baxter 2021	1	2017	1	2445	6.0%	1.21 [0.08, 19.39]
Etezadi 2011	2	85	34	557	58.4%	0.37 [0.09, 1.57]
Smith 2009	1	38	5	50	28.0%	0.24 [0.03, 2.17]
Tay 2022	1	21	1	14	7.6%	0.65 [0.04, 11.33]
**Total (95% CI)**		**2161**		**3066**	**100.0%**	**0.41 [0.15, 1.11]**
Total events	5		41			

Heterogeneity: Chi² = 0.93, df = 3 (P = 0.82); I² = 0%
Test for overall effect: Z = 1.75 (P = 0.08)

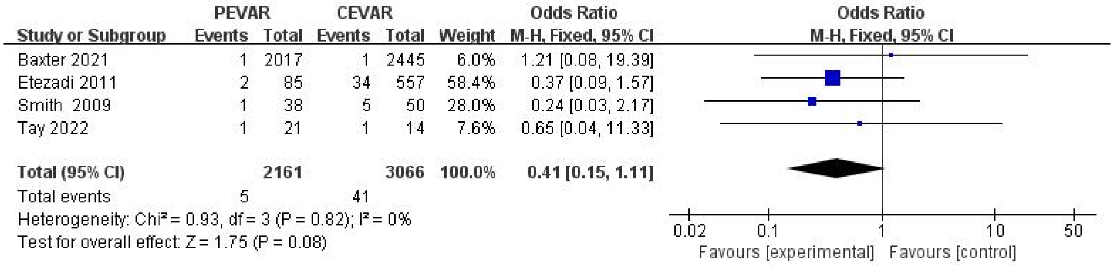

Figure26 Forest plot of literature on Groin infection with a follow-up time of 6 months or more

Study or Subgroup	PEVAR Events	PEVAR Total	CEVAR Events	CEVAR Total	Weight	Odds Ratio M-H, Fixed, 95% CI
Akbulut 2022	2	65	14	87	53.5%	0.17 [0.04, 0.76]
Baxter 2021	1	2017	1	2445	4.2%	1.21 [0.08, 19.39]
Etezadi 2011	1	85	3	557	3.6%	2.20 [0.23, 21.38]
Hahl 2023	1	257	5	186	26.6%	0.14 [0.02, 1.22]
Smith 2009	0	38	2	50	9.9%	0.25 [0.01, 5.40]
Tay 2022	3	21	0	14	2.3%	5.49 [0.26, 114.91]
**Total (95% CI)**		**2483**		**3339**	**100.0%**	**0.41 [0.18, 0.90]**
Total events	8		25			

Heterogeneity: Chi² = 7.88, df = 5 (P = 0.16); I² = 37%
Test for overall effect: Z = 2.22 (P = 0.03)

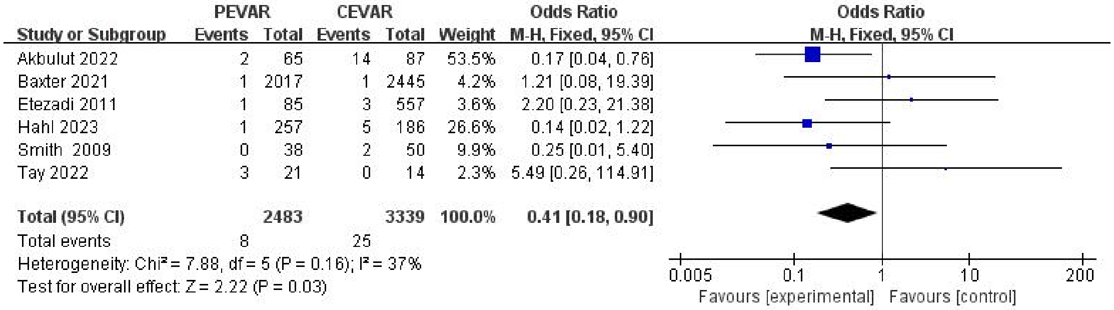

Figure27 Forest plot of literature on Lymphocele with a follow-up time of 6 months or more

Study or Subgroup	PEVAR Events	PEVAR Total	CEVAR Events	CEVAR Total	Weight	Odds Ratio M-H, Fixed, 95% CI
Akbulut 2022	0	65	6	87	73.5%	0.10 [0.01, 1.73]
Nelson 2014	0	101	1	50	26.5%	0.16 [0.01, 4.06]
**Total (95% CI)**		**166**		**137**	**100.0%**	**0.11 [0.01, 1.00]**
Total events	0		7			

Heterogeneity: Chi² = 0.06, df = 1 (P = 0.80); I² = 0%
Test for overall effect: Z = 1.96 (P = 0.05)

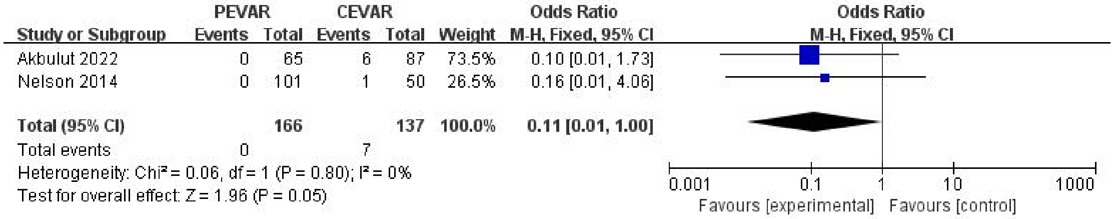

Figure28 Forest plot of literature on Acute kidney injury with a follow-up time of 6 months or more

Study or Subgroup	PEVAR Events	PEVAR Total	CEVAR Events	CEVAR Total	Weight	Odds Ratio M-H, Fixed, 95% CI
Nelson 2014	3	101	1	50	55.8%	1.50 [0.15, 14.80]
Tay 2022	3	21	1	14	44.2%	2.17 [0.20, 23.25]
**Total (95% CI)**		**122**		**64**	**100.0%**	**1.79 [0.35, 9.33]**
Total events	6		2			

Heterogeneity: Chi² = 0.05, df = 1 (P = 0.83); I² = 0%
Test for overall effect: Z = 0.70 (P = 0.49)

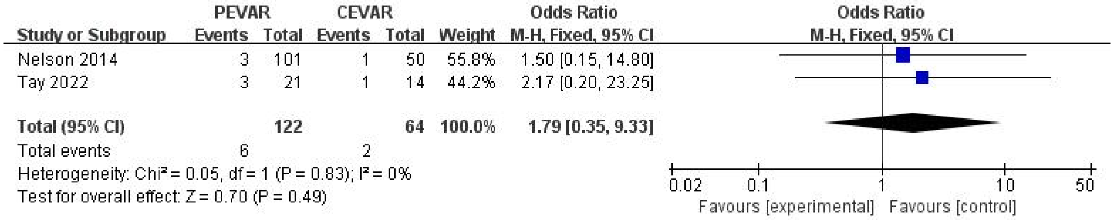

Figure29 Forest plot of literature on Lower extremityrevascularization with a follow-up time of 6 months or more

Study or Subgroup	PEVAR Events	PEVAR Total	CEVAR Events	CEVAR Total	Weight	Odds Ratio M-H, Fixed, 95% CI
Akbulut 2022	3	65	3	87	22.0%	1.35 [0.26, 6.94]
Baxter 2021	0	2017	3	2445	28.5%	0.17 [0.01, 3.35]
Nelson 2014	3	101	2	50	23.4%	0.73 [0.12, 4.54]
Tay 2022	0	21	2	14	26.1%	0.12 [0.01, 2.62]
**Total (95% CI)**		**2204**		**2596**	**100.0%**	**0.55 [0.21, 1.46]**
Total events	6		10			

Heterogeneity: Chi² = 2.81, df = 3 (P = 0.42); I² = 0%
Test for overall effect: Z = 1.20 (P = 0.23)

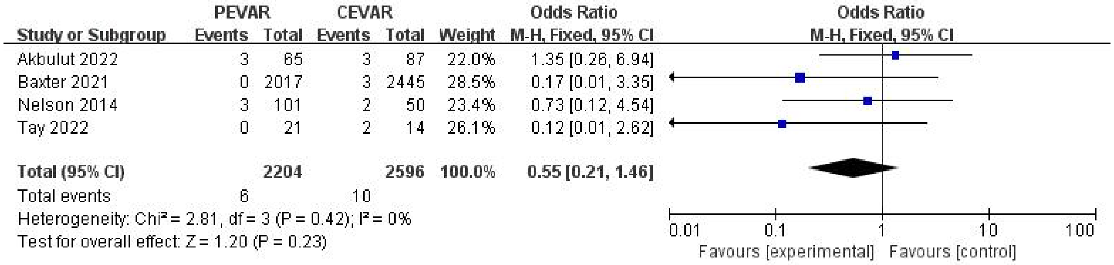

Figure30 Forest plot of literature on Deep venous thrombosis with a follow-up time of 6 months or more

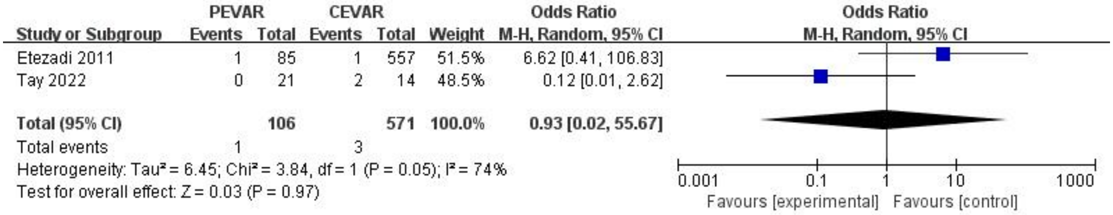

Figure31 Forest plot of literature on Pseudoaneurysm with a follow-up time of 6 months or more

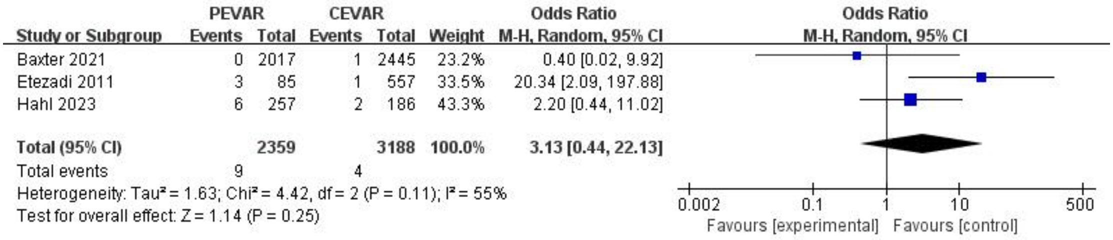

Figure32 Results of sensitivity analysis for Hospital length of stay

Results of sensitivity analysis of Hospital length of stay

Deletion of literature	*P* 值	*I* 2 (%)	95%*CI*	Effect size P-value
Sahin 2020	<0.00001	91	-1.82 [-2.70, -0.94]	<0.0001
Akbulut 2022	<0.00001	90	-1.67 [-2.60, -0.73]	0.0005
Altoijry 2023	<0.00001	91	-1.68 [-2.51, -0.86]	<0.0001
Baxter 2021	<0.00001	88	-1.69 [-2.66, -0.72]	0.0006
Hahl 2023	<0.00001	81	-2.00 [-2.81, -1.18]	<0.00001
Taku 2015	<0.00001	91	-1.67 [-2.54, -0.80]	<0.0002
Tay 2022	<0.00001	91	-1.77 [-2.60, -0.94]	<0.0001
Thurston 2019	<0.00001	90	-1.96 [-2.94, -0.97]	<0.0001
Wu 2022	<0.00001	87	-1.47 [-2.28, -0.66]	0.0004

Figure33 **Results of sensitivity analysis for operative time**

Results of sensitivity analysis of operative time

Deletion of literature	p-value	I^2 (%)	95%*CI*	Effect size P-value
Sahin 2020	＜0.00001	*94*	-38.78 [-47.61, -29.94]	＜0.00001
Akbulut 2022	＜0.00001	*95*	-42.75 [-52.19, -33.32]	＜0.00001
Howell 2002	＜0.00001	*93*	-37.32 [-45.66, -28.97]	＜0.00001
Kauvar 2016	＜0.00001	*94*	-43.96 [-53.25, -34.67]	＜0.00001
Lin 2018	＜0.00001	*95*	-42,71 [-52.29, -33.12]	＜0.00001
Nelson 2014	＜0.00001	*95*	-40.96 [-50.17, -31.74]	＜0.00001
Siracuse, 2018	＜0.00001	*95*	-43.26 [-55.56, -30.95]	＜0.00001
Taku 2015	＜0.00001	*95*	-39.59 [-48.68, -30.50]	＜0.00001
Tay 2022	＜0.00001	*93*	-35.78 [-43.83, -27.73]	＜0.00001
Torsello 2003	＜0.00001	*95*	-42.23 [-51.41, -33.05]	＜0.00001
Wu 2022	＜0.00001	*95*	-43.78 [-55.35, -32.21]	＜0.00001

Figure34 **Results of sensitivity analysis for Estimated blood loss**

Results of sensitivity analysis of Estimated blood loss

Deletion of literature	p-value	I^2 (%)	95%*CI*	Effect size P-value
Christopher 2017	＜0.00001	*92*	-67.40 [-105.57, -29.22]	0.0005
Howell 2002	＜0.00001	*100*	-97.04 [-259.69, 65.60]	0.24
Lin 2018	＜0.00001	*100*	-144.82 [-314.34, 24.71]	0.09
Nelson 2014	＜0.00001	*100*	-138.38 [-301.41, 24.65]	0.10
Siracuse 2018	＜0.00001	*100*	-149.19 [-322.41, 24.03]	0.09
Wu 2022	＜0.00001	*100*	-150.79 [-313.97, 12.40]	0.07

Figure35 Results of sensitivity analysis for Stay of postoperative

Results of sensitivity analysis of Stay of postoperative

Deletion of literature	p-value	I^2 (%)	95%*CI*	Effect size P-value
Christopher 2017	＜0.00001	*94*	-0.90 [-1.60, -0.21]	0.01
Kauvar 2016	0.001	*85*	-1.25 [-1.61, -0.89]	＜0.00001
Lin 2018	＜0.00001	*97*	-0.81 [-1.44, -0.17]	0.01
Siracuse 2018	＜0.00001	*97*	-1.06 [-1.91, -0.21]	0.01

Figure36 Results of sensitivity analysis for Patients requiring ICU stay

Results of sensitivity analysis of Patients requiring ICU stay

Deletion of literature	p-value	I^2（%）	95%*CI*	Effect size P-value
Altoijry 2023	0.008	*86*	0.23 [0.03, 1.73]	0.16
Christopher 2017	0.07	*70*	0.17 [0.04, 0.62]	0.008
Dipankar 2017	0.39	*0*	0.46 [0.21, 1.02]	0.06

Figure37 Results of sensitivity analysis for Deep venous thrombosis

Results of sensitivity analysis of Deep venous thrombosis

Deletion of literature	p-value	I^2（%）	95%*CI*	Effect size P-value
Dominique 2015	0.16	*42*	0.81 [0.18, 3.64]	0.78
Etezadi 2011	0.03	*67*	0.88 [0.24, 3.22]	0.84
Kauvar 2016	0.24	*28*	1.86 [0.52, 6.56]	0.34
Lin 2018	0.02	*71*	1.02 [0.26, 4.00]	0.88
Tay 2022	0.03	*65*	1.48 [0.43, 5.17]	0.53
